# CXCL9-driven immune rewiring and tumor-intrinsic ATR signaling inhibition restore radiotherapy response in lung cancer

**DOI:** 10.1186/s13046-026-03834-z

**Published:** 2026-09-22

**Authors:** Sergio Leon, Nerea Otegui, Miriam Redrado, Eric Michel Marchena-Perea, Elisabeth Guruceaga, Flavia Castro, Luis M. Montuenga, María José Oliveira, José Javier Aristu, Diego Serrano, Alfonso Calvo

**Affiliations:** 1 Program in Solid Tumors, CIMA-University of Navarra, Cancer Center Clinica Universidad de Navarra (CCUN), Pamplona, Spain; 2 CIBERONC, ISCIII, Madrid, Spain; 3 Department of Pathology, Anatomy and Physiology, School of Medicine, University of Navarra, Pamplona, Spain; 4 IDISNA, Pamplona, Spain; 5 Bioinformatics Platform, CIMA-University of Navarra, Cancer Center Clinica Universidad de Navarra (CCUN), Pamplona, Spain; 6 i3S - Instituto de Investigação e Inovação em Saúde, Universidade do Porto, Porto, Portugal; 7 INEB - Instituto de Engenharia Biomédica, Universidade do Porto, Porto, Portugal; 8 ICBAS - Instituto de Ciências Biomédicas Abel Salazar, Universidade do Porto, Porto, Portugal; 9 Department of Radiation Oncology, Clinica Universidad de Navarra, Pamplona, 31008 Spain

**Keywords:** Radioresistance, Isogenic cell models, ATR, CXCL9, Immunosuppression, Innate immunity

## Abstract

**Background:**

Non-small cell lung cancer (NSCLC) accounts for ~ 85% of lung cancers and is associated with poor outcomes in advanced stages. Radiotherapy (RT) is central to NSCLC treatment, yet locoregional relapse remains frequent. Increasing evidence indicates that, beyond tumor-intrinsic adaptations, the tumor microenvironment (TME) and immune contexture critically influence response to irradiation. However, most radioresistance models rely on human cell lines unsuitable for immunocompetent systems, limiting insight into immune-tumor interactions.

**Methods:**

We generated radioresistant (RR) isogenic variants of murine lung adenocarcinoma cell lines and investigated RT resistance using transcriptomics, 3D co-culture systems, and syngeneic mouse models.

**Results:**

RR clones showed stable adaptations to DNA damage, including enhanced clonogenic survival, reduced immunogenic cell death, and constitutive activation of DNA damage response pathways, particularly ATR signaling. High ATR levels in NSCLC patients were significantly associated with immunosuppression. Transcriptomics revealed altered DNA repair and downregulation of type-I interferon and innate immune pathways. In vivo, RR tumors exhibited an immunosuppressive TME with increased T regulatory cells, M2-like macrophages, and myeloid-derived suppressor cells, alongside reduced cytotoxic T cell recruitment. In 3D co-cultures, immunosuppressive cells protected cancer cells from RT-induced cytotoxicity. Notably, RR tumors regained RT sensitivity in immunodeficient mice, highlighting the role of immune suppression in radioresistance. Therapeutically, activation of innate immunity using TLR3-TLR7/8 agonists (poly(I:C)+R848) restored antitumor responses and improved RT efficacy, partly via CXCL9/10-mediated immune recruitment. ATR inhibition with ceralasertib further sensitized RR tumors to RT.

**Conclusion:**

These findings identify cancer cell-intrinsic ATR signaling and immune suppression as key drivers of radioresistance and support combined therapeutic strategies to prevent locoregional relapse in NSCLC after radiotherapy.

**Supplementary Information:**

The online version contains supplementary material available at https://doi.org/10.1186/s13046-026-03834-z.

## Background

Non-small cell lung cancer (NSCLC) accounts for approximately 85% of all lung cancer cases, and despite current therapeutic approaches, survival remains very poor in advanced stages. Patients with operable stage I and II disease are treated primarily with surgical resection, often followed by adjuvant chemotherapy or immunotherapy. For medically inoperable early-stage patients, conventional radiotherapy (RT) or stereotactic body radiotherapy (SBRT) is the standard approach. In stage III, NSCLC patients with unresectable disease are managed with concurrent chemoradiotherapy followed by consolidation immunotherapy (e.g., with anti-PD-L1) [1]. In advanced or metastatic stages, systemic chemotherapy, targeted therapies for oncogene-driven tumors, and immune checkpoint inhibitors (ICIs) have become standard treatments, with RT used frequently as a palliative strategy [2, 3]. Although RT can be highly effective, locoregional progression develops in a substantial proportion of NSCLC patients and is associated with poor prognosis, suboptimal local and distant control, and treatment failure. In patients treated with definitive chemoradiotherapy for locally advanced NSCLC, local failure rates in the thorax can be as high as 30–50% at two years, with isolated locoregional recurrences seen in about 20–30% of cases. Therefore, locoregional failure remains common despite improvements in therapy and negatively impacts overall treatment outcomes [4, 5].

RT induces cancer cell death through DNA double-strand breaks (DSBs) and other lethal genomic or cellular damage [6]. Irradiation also affects the tumor microenvironment (TME), which plays a critical modulatory role in determining RT outcomes. Irradiation can elicit immunogenic cell death (ICD), promote the release of tumor-associated antigens (TAAs) and damage-associated molecular patterns (DAMPs), upregulate MHC-I expression, activate the cGAS-STING pathway and type I interferon response, and recruit dendritic cells (DCs) and T lymphocytes to the irradiated site, thereby enhancing anti-tumor immune responses [7]. Nonetheless, persister cells after repeated irradiation cycles can adapt and modify the TME towards a more immunosuppressive phenotype. This can lead to increased fibrosis and stromal remodeling, recruitment of myeloid-derived suppressor cells (MDSCs) and T regulatory cells (Treg cells), as well as polarization of macrophages to acquire M2-like traits [7, 8]. These factors will facilitate local and distant recurrence.

To investigate cancer cell intrinsic mechanisms of radioresistance, several studies have generated radioresistant (RR) human NSCLC cell lines by exposing them to repeated rounds of ionizing radiation in vitro. Commonly used parental cell lines include A549, H520 and H460, which were subjected to different irradiation protocols and doses to obtain their corresponding isogenic RR clones [9]. These RR populations generally showed increased clonogenic survival and elevated expression of epithelial-mesenchymal transition (EMT) markers. Transcriptomic alterations included upregulation of DNA damage response (DDR) genes, metabolic adaptations toward a “pseudo-hypoxic” phenotype, and increased secretion of immunosuppressive cytokines [10]. However, because these models are derived from human cell lines, they cannot be implanted in immunocompetent mice, limiting insights into how RR cancer cells modulate immune populations in vivo. Therefore, appropriate transplantable isogenic cancer models of radioresistance are needed to assess the crosstalk between cancer cells and the TME.

In the present study, we have used the well characterized murine lung adenocarcinoma cell lines Lacun3 and CMT167, to generate their radioresistant isogenic counterparts (Lacun3 RR and CMT167 RR). These cells can be inoculated in syngeneic immunocompetent mice, where we have found that immunosuppressive cell populations play a determinant role in resistance to RT. Through transcriptomic and functional 3D co-cultures and in vivo assays we have demonstrated the role of M2-like macrophages and Treg cells in protecting cancer cells from irradiation-mediated cell death, and downregulation of STING/IFN-I pathways and CXCL9/10 signaling in RR tumors. Moreover, radioresistance increases phospho (p)-ATR levels in cancer cells and ATR mRNA levels associate with immunosuppression in NSCLC patients. In our in vivo models, a combination of poly(I:C)+R848 (resiquimod) with the ATR inhibitor ceralasertib, resensitizes tumors to RT. We also found that CXCL9 plays a key role in chemotaxis of splenocytes that migrate towards tumor cells in treated animals, revealing a key role of this chemokine in overcoming immune-mediated radioresistance.

## Methods

### Cell culture

Murine lung adenocarcinoma cell lines Lacun3 (L3 from now on), previously developed and characterized in our Laboratory [11, 12] and CMT167 [13, 14] (CMT from now on), kindly donated by Dr. Paola Allavena (Department Immunology, IRCCS Humanitas Research Hospital, Milan, Italy) were used in this study. To generate their respective RR variants (L3^RR^ and CMT^RR^), several rounds of RT were applied, following the experimental procedure described in Fig. S1A. Both parental and L3^RR^ cells were cultured in RPMI medium 1640 (#61870010, Gibco) supplemented with 10% of FetalClone III (#10570083, Thermo Fisher Scientific) and 1% of penicillin/streptomycin (#15140122, Gibco) in standard culture conditions. CMT and its RR variant were cultured in DMEM medium (#41966029, Gibco) supplemented with 10% of FetalClone III and 1% of penicillin/streptomycin. Murine Raw246.7 macrophages were also used and cultured in supplemented RPMI, as explained before.

### CXCL9 overexpression of L3^RR^ cell lines

Overexpression of CXCL9 was carried out by infection of the L3^RR^ and CMT^RR^ cells with viral particles containing the lentiviral plasmid pLV encoding the sequence of murine CXCL9 (VectorBuilder, VB250825-1133ckd). 25,000 cells were infected by adding virus and polybrene (8 µg/mL) for 72 h. Selection of infected cells was carried out by adding 5 µg/mL of puromycin (Sigma, #P7255) to the media every 48 h, for 7 days. A pLV plasmid containing a scramble sequence from VectorBuilder was used as control.

### Irradiation protocols

For in vivo irradiation of tumor-bearing mice, we used the Small Animal Radiation Research Platform (SARRP, Xstrahl). Mice were anesthetized with 40 µL of anesthesia (450 µL of physiological serum (#606973.8, Braun), 450 µL of Ketamidor (#280393.7, etViva) and 100 µL of Rompun (#572126.2, Elanco)). Anesthetized mice were individually placed on the irradiation platform and subcutaneous tumors were immobilized using plastic clamps. Dosimetry was adjusted to deliver the prescribed dose at the tumor isocenter. For this purpose, a mouse CT scan image was acquired using the SARRP system, and the reconstructed images were imported into Muriplan software (Xstrahl) for dose calculation. The resulting tumor coordinates and irradiation time were then applied uniformly to all mice in the experiment. A 10 × 10 mm collimator was used, and the radiation exposure time was adjusted to deliver the desired dose. For irradiation of cells in vitro, the plates were moved to the SARRP irradiator and irradiated at different doses depending on the assay.

### qPCR and western blotting

RNA was isolated using the NucleoSpin^®^ RNA method (#740955.25, Macherey-Nagel) following the manufacture’s protocol. Upon extraction, Nanodrop (Thermo Scientific) was used for RNA quantification and assessment of RNA quality. Nucleic acids were stored at -80 °C until use. For RNA retrotranscription, the Applied Biosystems™ High-Capacity cDNA Reverse Transcription Kit (#4374967, Applied Biosystems) was used following manufacturer’s instructions in a Peltier Thermal Cycler (PTC-100, Bio-Rad Laboratories). For real time quantitative PCR (qPCR), a master PCR mix containing SYBR-Green (#4309155, Applied Biosystems) was used. Sequences of the forward (FW) and reverse (RV) primers for each gene are listed in Table S1. PCRs were run in a QuantStudio3 thermal cycler (Thermo Fisher Scientific). The PCR protocol was as follows: 2 min at 50 °C, 10 min at 95 °C, 15 s at 95 °C (for 40 cycles) and 1 min at 60 °C. Analysis was carried out with QuantStudio™ Design & Analysis Software.

For western blotting, cell pellets were washed with PBS and lysed with RIPA Buffer (1 mL of Tris 1 M (pH 7.4), 0.8775 g NaCl (pH 7.4), 1 mL of Triton-X-100, 1 g of sodium deoxycholate, 0.1 g of sodium dodecilsulfate (SDS) and H_2_Od to 100 mL) supplemented with Complete™, EDTA-free Protease Inhibitor Cocktail (#11836170001, Roche) and PhosSTOP™ (#4906845001, Roche). Proteins were quantified with the BCA kit (#23225, Thermo Fisher) and NuPAGE™ 4–12% Bis-Tris protein gels (#NP0321BOX, Invitrogen) were used for the electrophoresis in MOPS SDS Running Buffer (#NP0001-02, Invitrogen). Electrophoresis was carried out at 75 V for 10 min and 130 V for 2 h. Transference was performed at 110 V for 1 h and Ponceau Solution (#P7170, Sigma Aldrich) was used for quality control. Membranes were blocked with 5% BSA for 1 h and incubated overnight at 4 °C with primary antibodies. The list of primary and secondary antibodies is shown in Table S2. Bands were visualized and quantified with Lumi-Light kit (#32109, Thermo Scientific), based on a chemiluminescence process in an Odyssey Fc (LI-COR).

### Clonogenic assays

For clonogenic assays, 750 L3 or L3^RR^ cells were cultured in standard conditions. Cells were either treated with radiation doses of 2 Gy, 4 Gy, 6 Gy or 8 Gy, or left untreated (controls). Medium was renewed every 48 h and, seven days after irradiation, the medium was removed and cells were fixed with 4% formaldehyde (#252931.1211, PanReac AppliChem) and stained with Crystal Violet (#V5265, Sigma Aldrich). For CMT and CMT^RR^ cells, a similar protocol was carried out, but cells were irradiated at 8, 10, 12, 15 or 20 Gy doses. Clonogenic capability was calculated by measuring the percentage of area covered with Crystal Violet using QuPath software (version 0.5.1).

### Cytotoxicity assays and cell proliferation

Cells (1,000 per well) were seeded in 96-well plates and irradiated (6 Gy) 24 h after seeding. VE-821 (#HY-14731, MedChemExpress), ceralasertib (#HY-19323, MedChemExpress), S6Ki (#PF-4708671, MedChemExpress) or P38i (#LY2228820, Selleckchem) were added 2 h after irradiation. Absorbance data was measured 4 days after treatments using the MTS assay (#G3581, Promega). Similarly, to assess basal cell proliferation, absorbance of untreated cells was carried out 24, 48 and 72 h after seeding. Data were normalized with those obtained at day 0.

### Immunofluorescence

70,000 cells were seeded in 4-well Culture Slide (#354104, Corning), irradiated (6 Gy) 24 h after seeding and fixed with 4% formaldehyde for 15 min at room temperature 6 h post-irradiation. After blockade of unspecific binding sites and permeabilization (2% BSA and 0.3% Triton X-100 in PBS 1X) for 30 min, samples were incubated with anti-phospho-histone H2A.X (#2577, Cell Signaling) diluted 1:500 in 2% BSA at 4 °C overnight. Next, Alexa Fluor 594 anti-rabbit antibody (#A11037, Invitrogen) was added for 2 h (1:200 in 2% BSA). Finally, cells were stained with DAPI (#FP1490, Akoya) and mounted with Prolong diamond anti-fade mountant solution (#P36961, Invitrogen). Slides were scanned with an Axio Imager M1 microscope (ZEISS), and QuPath software was used to analyze fluorescence intensity and loci counting.

### Apoptosis assay

Cells (50,000) were seeded in 60 mm TC-treated culture plates (#430166, Corning). Next day, cells were either drug-treated (ceralasertib), irradiated (8 and 15 Gy) or left untreated (controls). For the staining, cells were collected 7 days after treatments, trypsinized, washed with PBS and incubated with anti-mouse APC-Annexin V (#ANXVDY-200T, Immunostep), diluted 1:100 in Annexing binding buffer (#556454, BD Bioscience) for 15 min. SYTOX-Green (#S34860, Invitrogen, 1:1000) was added before acquisition of the cells in a CytoFlex S cytometer and analysis using CytExpert software (version 2.5.0.77).

To study the effect of ceralasertib, poly(I:C) (pIC) (#tlrl-pic, Invivogen) and resiquimod (R848) (#tlrl-r848-10, Invivogen) in vitro, 100,000 L3 or L3^RR^ cells were seeded in 6-well cell culture plates (#CLS353224, Corning). The next day, cells were irradiated (8 Gy) or left untreated, and 2 h post irradiation, ceralasertib (1 µM) or a mix containing pIC+R848 (5 µg/mL each) was added to the media. 2 days after treatments, cells were collected and stained for apoptosis as previously explained.

### Estimation of immunogenic cell death

To assess immunogenic cell death (ICD), levels of calreticulin in the plasma membrane were evaluated. Cells (100,000) were seeded in 6-well cell culture plates. 24 h later, cells were either irradiated (8 Gy) or not (controls). 24 h post-irradiation, they were collected and stained with ViaKrome 808 (1:100) (#C36628, Beckman Coulter), for 30 min in cold PBS at 4 °C in darkness. Then, anti-calreticulin antibody (#ab2907, Abcam) was diluted (1:100) in FACS buffer (2.5 mmol/L EDTA and 5% FetalClone in PBS) for 30 min at 4 °C in darkness. Next, cells were fixed with 4% formaldehyde for 15 min and then, an Alexa Fluor 594 anti-rabbit antibody diluted 1:250 in FACS buffer was applied. Oxaliplatin (0.3 mM) was used as positive control. Cells were acquired in a CytoFlex LX cytometer and analyzed with CytExpert software.

### In vivo experiments

Eight-week-old female Balb/c, C57BL/6J and athymic nude mice (from Envigo) were kept in a pathogen-free environment at CIMA’s animal facility. All animal experiments were approved by the Ethical committee for the experimentation with animals (CEEA) of our institution (ref. #082 − 23). L3 and L3^RR^ cells (10^6^) were subcutaneously injected into the right limb of Balb/c or athymic nude mice. CMT (10^5^) and CMT^RR^ (2 × 10^5^) cells were subcutaneously injected into the right limb of C57BL/6J or athymic nude mice. Cells were injected in 100 µL of complete RPMI medium (L3 and L3^RR^) or complete DMEM medium (CMT and CMT^RR^). Mice were monitored by measuring the tumor size with a digital caliper every 48–72 h. When tumor volume reached 50 mm^3^, mice were randomized and treated.

The RT scheme for L3- and L3^RR^-tumor bearing mice was 8 Gy in three consecutive days (3 × 8 Gy), and for CMT- and CMT^RR^-tumor bearing mice, two different schemes in five consecutive days were tested (5 × 5 Gy and 5 × 8 Gy). Radiation treatments were performed with the SARRP, as described before.

Experiments to deplete CD8^+^ cells (#BE0061, anti-mouse CD8α, clone 2.43, BioXcell), CD4^+^ cells (#BE0003-1, anti-mouse CD4, clone GK1.5, BioXcell), GR1^+^ cells (#BE0075, anti-mouse Ly6G/Ly6C (Gr-1), clone RB6-8C5, BioXcell) and macrophages (#BE0213, anti-mouse CSF1R, clone AFS98, BioXcell) were carried out by intraperitoneal injection of 100 µg of αCD8 and αCD4 depleting antibodies, 200 µg of αGR1 and 500 µg of αCSF1R every 2 days, starting one-day prior RT treatments. A scheme showing the experimental design is shown in Fig. S1B. To study the effects of the ATRi ceralasertib, L3^RR^-tumor bearing mice were administered with 70 mg/kg of the drug, by oral gavage (o.g.), in vehicle [10 vol% DMSO (#D8418, Sigma-Aldrich), 40 vol% polyethylene glycol 400 (#91893, Sigma-Aldrich), 5 vol% Tween-80 (#P1754, Sigma Aldrich), 45 vol% Saline solution (#607189.2, Braun)].

Experiments to overcome resistance to RT were performed by intratumoral (i.t.) administration of a mix containing pIC and R848 (25 µg each) every 2 days (four doses), starting the day prior to the corresponding RT schemes. Ceralasertib was administered as explained before, 2 h prior RT. Control groups were treated with their corresponding vehicles.

To assess toxicity, spleen and kidneys were obtained from mice and processed for histopathological examination. In addition, plasma was obtained upon centrifugation of blood (1,200 rpm, 30 min, 4 °C) to quantify liver enzymes in a Cobas c 311 analyzer (Roche).

### Flow cytometry

Subcutaneous tumors and spleens were excised, mechanically and enzymatically disaggregated by incubation with DNAse I (#10104159001, 50 µg/mL, Roche) and collagenase D (#11088866001, 1 mg/mL, Roche) for 15 min at 37 °C, in agitation. Samples were filtered through 70 μm Cell Strainers (#CLS352350, Corning) and centrifuged at 1,500 rpm for 5 min. Then, erythrocytes were lysed with ACK Lysing Buffer (#A1049201, Gibco) and cells were centrifuged at 1,500 rpm for 5 min. Samples were processed in 35% Percoll solution [35% Percoll (#17089101, Sigma-Aldrich), 62% RPMI-1640, 3% 10X PBS (#5521-031-CV, Corning) and 2.2 mg heparin (#654754 Hospira)] and centrifuged at 1,500 rpm for 15 min. For cell viability, ViaKrome 808 (1:100) diluted in cold PBS for 30 min at 4 °C was used. For extracellular staining, cells were incubated with fluorochrome-conjugated antibodies diluted in FACS Buffer for 30 min at 4 °C. For intracellular staining, samples were incubated with Fixation/Permeabilization buffer (#00-5523-00, Invitrogen) for 30 min at 4 °C and incubated with antibodies diluted in permeabilization buffer (#00-8333-56, Invitrogen) for 30 min at 4 °C. Samples were acquired in a CytoFlex LX cytometer (Beckman Coulter) and data were analyzed in CytExpert software (version 2.5.0.77). Antibodies are shown in Table S3 and gating strategies are shown in Fig. S2 and S3.

For flow cytometry of PD-L1, 50,000 L3 and L3^RR^ cells were seeded in 6-well cell culture plates. One day before the acquisition, IFNγ (500 U/µL) (#315-05 Peprotech) was added to the media. Cells were then collected, washed with PBS and stained with anti-PD-L1-PE (Invitrogen, 12-5982-81) diluted 1:500 in FACS Buffer for 30 min at room temperature. Cells were resuspended in FACS Buffer until acquisition in the CytoFlex Dx cytometer (Beckman Coulter).

### Isolation and differentiation of murine immune populations

Murine naïve CD4^+^ T cells and monocytes were isolated from the spleens and bone marrows of Balb/c and C56BL/6J mice. The spleen was mechanically grinded and passed through a 70 μm cell strainer. The splenocytes were lysed with ACK lysing buffer for 5 min and resuspended in RPMI media (Supplemented with with MEM Non-Essential Aminoacids (#11140-050, Gibco), HEPES Buffer (#15630-056, Gibco), Na-Pyruvate (#11360-070, Gibco) and Fungizone (#SV30078.01, Cytiva)). CD4^+^ cells were negatively immunoseparated with the EasySep Mouse Naïve CD4^+^ T Cell Isolation Kit (#19765A, STEMCELL Technologies). CD4^+^ T cells (50,000) were seeded in U-bottom 96-well plates (#3879, Corning), previously treated with 1 µg/mL αCD3 (#553057, BD Pharmingen) and 0.5 µg/mL αCD28 (#553295, BD Pharmingen) antibodies in PBS, prepared one day before seeding. Cells were cultured with 5 ng/mL TGFβ (#Rcyc-htgfb1, InvivoGen) and IL2 100 U/mL Proleukin (#CN70389, Novartis) for 5 days. Cytokines were renewed at day 3 of differentiation. In these experiments, ceralasertib was added for 5 days at a concentration of 10 nM.

Bone marrow was extracted from both hindlimbs and mechanically disrupted by passing it through a 1 mL syringe (#303 − 172, BD). Cells were transferred into a microtube containing 300 µL of 2.5% FBS in PBS. After centrifugation, cell suspension was lysed with ACK lysing buffer for 5 min and resuspended in complete RPMI medium. Monocytes were negatively isolated with the EasySep™ Mouse Monocyte Isolation Kit (#19861, STEMCELL Technologies). To induce M2-like macrophage differentiation, 10^6^ monocytes were seeded in 6-well plates and cultured in complete RPMI medium supplemented with 25 ng/mL of M-CSF (#315-02, Peprotech) for 4 days. Finally, to allow polarization to M2-like macrophages, fresh RPMI containing 100 U/mL of IL4 (#214 − 14, Peprotech) was added for an additional day. Immunoseparations were performed using the EasyEights EasySep Magnet (#18103, STEMCELL Technologies).

### Three-dimensional coculture of murine immune cells with tumor cells

For 3D co-culture experiments, agarose micromolds (Microtissues 3D Petri dish micromolds, #Z764019, Merck) were used. For mold casting, sterile agarose (#50004, SeaKem LE Agarose, Lonza) was dissolved in sterile saline solution (0.9% NaCl in water) to a final concentration of 2% agarose. This solution was melted and added to the microTissue 3D Petri Dish micromolds. When solidified, micromolds were introduced in individual wells of 12-well untreated culture plates (#734–2778, Avantor) and hydrated with the corresponding complete medium for at least 2 h.

Tumor spheroids (TSp) and tumor-immune spheroids (TImS) were generated by seeding 2,500 tumor cells alone or in combination with Treg cells and M2-like macrophages, at a ratio 10:50:40 as we have previously described [15]. Then, wells were filled up to 2 mL with the corresponding complete medium (with 500 U/mL IL2 in the case of TImS). For TImS visualization, 10^6^ cells were resuspended in 1 mL of PBS and stained with 1 µL of CellTrace Violet for tumor cells (5 mM prepared in DMSO; #C34557, Thermo Fisher Scientific), CellTrace Far Red for M2-like macrophages (1 mM prepared in DMSO; #C34572, Thermo Fisher Scientific) or CellTrace CFSE for Treg cells (5 mM prepared in DMSO; #C34554, Thermo Fisher Scientific) for 20 min at 37 °C. For irradiation of the TSp and TImS, plates were carefully moved to the SARRP irradiator and irradiated at 6 Gy.

### Immunohistochemistry and multispectral immunofluorescence (mIF)

Paraffin-embedded tissues were dewaxed by heating the sample (15 min at 60 °C), and then placed in xylene-containing jars for 15 min. After immersion in ethanol (absolute to 70%) slides were transferred to water and endogenous peroxidase was blocked by incubation with 3% H_2_O_2_ for 10 min. For immunohistochemistry, upon antigen retrieval, tissues were incubated with the primary antibodies diluted in Dako diluent (#S2022, Dako) at 4 °C overnight. Next, samples were incubated with their corresponding anti-mouse or anti-rabbit EnVision polymer (#K4003, Dako) for 30 min at room temperature. Finally, diaminobenzidine (#K368, Dako) was used to reveal the signal. Samples were counterstained with Harris hematoxylin and slides were mounted in DPX mounting solution (#06522, Sigma-Aldrich) and scanned in an Aperio CS2 scanner (Leica). Positive cells were quantified using QuPath (version 0.4.3). Details on antigen retrieval and specification of primary antibodies are shown in Table S4.

For mIF, the murine-specific kit from Akoya (#NEL840001KT) was used after adaptation of the protocol from the manufacturer to our experimental conditions following our previously published protocol [16]. Opals 520, 570 and 690, as well as spectral DAPI for nuclear staining, included in the kit, and opals 480 (#FP1500001KT), 620 (#FP1495001KT) and 780 (#FP1501001KT), were purchased from Akoya. First, a panel to study CD4^+^, CD8^+^, FOXP3^+^, PD-1^+^, F4/80^+^ and PD-L1^+^ cells was used. A second panel to study F4/80^+^, Arg1^+^ and CD86^+^ cells was also used to study macrophage polarization in poly(I:C)+R848 treated mice. Details on antibodies and experimental details can be found in Table S5.

Sample scanning, spectral unmixing and quantification of signals were conducted with the Vectra Polaris Automated Quantitative Pathology Imaging System (Akoya) and inform 2.4 (Akoya) and QuPath software was used for signal quantification. Data were given as the number of positive cells with a specific immunophenotype/total number of cells.

### Quantification of cytokine levels in plasma

To measure plasma levels of IL1β, CXCL16, IP10 (CXCL10), MCP1 (CCL2), MIG (CXCL9), IL25 and IL6 cytokines, a customized multiplex kit from AssayGenie was used. 45 µL of capture-bead-working solution were added to each well and the buffer was removed by using the flow-through Filter Plate Washer (#ACAM00001, AssayGenie) provided by the kit. Next, 45 µL of plasma were added and incubated on a shaker (700 rpm) for 1 h at room temperature. Then, samples were incubated with 25 µL of biotinylated antibody for 30 min, washed and incubated with 25 µL of streptavidin-PE for 20 min. Finally, samples were diluted in 200 µL of Reading Buffer and acquired in CytoFlex LX.

### Transwell chemotaxis assay

This assay was used to quantify splenocytes migration toward different stimuli using 6.5-mm Transwell inserts (24-well format), with 5-µm pore polycarbonate membranes (#3421, Corning). Tumors and spleens were excised and mechanically disaggregated as described before. Briefly, 50,000 tumor cells were seeded in complete RPMI medium alone or supplemented with 200 nM anti-mouse CXCL9/MIG (#HY-P990255, MIG-2F5.5; MedChemExpress) and anti-mouse CXCR3 (#AMG487; MedChemExpress). After 4 h, 500,000 splenocytes, previously tracked with CellTrace Violet as described earlier, were added on the inserts. After 20 h, supernatants in the lower chamber were collected and acquired in the CytoFlex LX cytometer. For absolute cell quantification, Precision Count Beads (#424902; BioLegend) were added immediately prior to acquisition.

### RNA and DNA sequencing

For transcriptomic analysis, two sets of samples were used: a) RNA isolated from L3 and L3^RR^ cells, either irradiated (6 Gy, 24 h) or not (n = 3 replicates per group); b) RNA extracted from surgically excised L3 or L3^RR^ tumors (irradiated or not; n = 6 per group) grown in Balb/c mice. After quality control using Tape Station (Agilent), samples were sequenced by Macrogen (South Korea). Truseq stranded mRNA Library + NovaSeq6000 150PE (150 × 2) with 30 M total reads/sample was performed, which consisted of readings of 150 bp pairs in 5’-3’ direction and vice versa.

For whole exome sequencing (WES), genomic DNA from L3 and L3^RR^ cells was used. A small fragment of a healthy Balb/c mouse tail DNA was also analyzed as background control. Genomic DNA was extracted using the NucleoSpin Tissue kit (#740952.50, Macherey-Nagel) following the kit’s instructions. Samples were sequenced by Macrogen using the SureSelectXT Library. Paired-end and 150 bp, with 20 M total reads/sample was performed.

### Bioinformatic and statistical analysis

RNA sequencing data analysis was performed using the following workflow: (1) the quality of the samples was verified using FastQC software (https://www.bioinformatics.babraham.ac.uk/projects/fastqc/) and the trimming of the reads with trimmomatic [17]; (2) alignment against the mouse reference genome (GRCm39) was performed using STAR [18]; (3) gene expression quantification using read counts of exonic gene regions was carried out with featureCounts [19]; (4) the gene annotation reference was Gencode vM35 for the in vitro experiment and vM33 for the in vivo experiment [20]; and (5) differential expression and statistical analysis was performed using R/Bioconductor [21].

Gene expression data was normalized with edgeR [22] and voom [23]. After quality assessment and outlier detection using R/Bioconductor [21], a filtering process was performed. Genes with read counts lower than 6 in more than 50% of the samples of all the studied conditions were considered as not expressed in the experiment under study. LIMMA [23] was used to identify the genes with significant differential expression between experimental conditions. Further functional and clustering analyses and graphical representations were performed using R/Bioconductor [21] and clusterProfiler [24]. The functional analyses included Gene Set Enrichment Analysis (GSEA) with MSigDB C2 collection of gene sets [24, 25]. Only genes with B > 0 and logFC > 0.5 or logFC<-0.5 were considered as differentially expressed genes (DEGs).

Analysis of mutations was conducted as follows: For DNA alignment, BWA-MEM algorithm (BWA/0.7.17-foss-2018b) using GRCm39 mouse genome as reference was used. The SAM files were converted to BAM files, which were then processed using the Mutect2 algorithm, contained in the Genome Analysis ToolKit (GATK) [26]. Finally, annotation and genome visualization were performed with ANNOVAR [18] and IGV program [27].

The correlation between ATR mRNA levels and the immunosuppressive score was performed in LUAD patients (*n* = 600) by interrogating data from The Cancer Genome Atlas (TCGA) database. The immunosuppressive score was obtained by calculating the mean expression of the following genes: *PD-L1*, *PDCD1 (PD-1)*, *CTLA4*, *LAG3*, *TIGIT*, *TGFB1* and *IL10.* Correlation index was calculated with the Spearman correlation test.

Normality of data was assessed with the Shapiro-Wilk test. Statistical comparisons between two groups were performed with Student’s t-test (parametric) or U-Mann Whitney (non-parametric). Comparison between multiple groups were estimated with the two-way ANOVA followed by Bonferroni post-hoc correction test. The statistical tests used are described in each Figure legend. Data were plotted and analyzed with GraphPad software (GraphPad Prism 8.0.1) and statistical significance was defined as *p* < 0.05 (*), *p* < 0.01 (**) and *p* < 0.001 (***).

## Results

### Radioresistant isogenic cancer cell clones show permanent adaptations to DNA damage response and particular sensitivity to ATR inhibitors

**Fig. 1:**
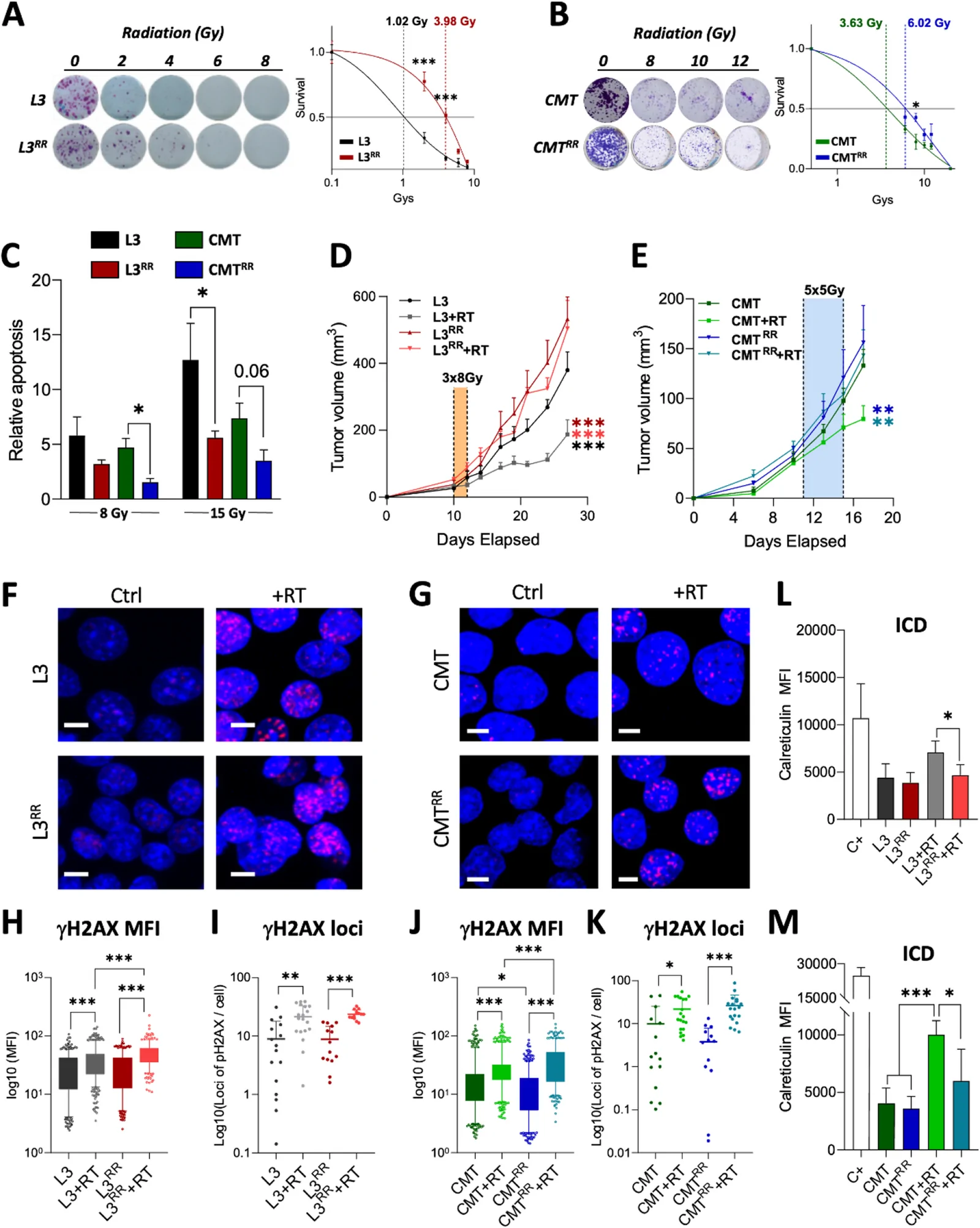
(**A**, **B**) Representative images of clonogenic assays and quantification of cell survival, using different radiation doses in L3, L3^RR^, CMT and CMT^RR^ cells. Survival analysis shows that RR cells tolerated better radiation than parental cells. **C** Apoptosis analyses show significantly less proportion of dead cells in RR cells than in parental cells upon RT treatment, thus validating radioresistance. **D**, **E** Validation of radioresistance in vivo. Tumor volumes over time reflect that irradiated tumors generated from RR cells continue growing at a similar rate than controls, unlike those derived from parental cells, where a significant decrease (*p*<0.001 for L3; *p*<0.01 for CMT cells) is observed. **F**, **G** Representative images of immunofluorescence for γH2AX in parental and RR cells, untreated or treated with RT. Scale bar: 5 µm. **H** to **K** Analysis of mean fluorescence intensity (MFI) and number of loci in the different conditions shown in Fig. 1**F**, **G**. Signal intensity is significantly increased in both parental and RR cells when treated with RT, but the signal in RR cells is significantly higher than that of parental cells (*p*<0.001). **L**, **M** Quantification of calreticulin in the plasma membrane to evaluate immunogenic cell death (ICD) of L3, L3^RR^, CMT and CMT^RR^ either irradiated or not. Oxaliplatin (0.3 mM) was used as positive control. Statistical significance was defined as *p*<0.05 (*), *p*<0.01 (**) and *p*<0.001 (***)

To establish the models of radioresistance in a context of complete immune system, we selected the aggressive and invasive murine lung adenocarcinoma cancer cell lines L3 and CMT. Radioresistant clones were obtained by several rounds of irradiation, selection of the persister cells and subsequent irradiation cycles (Fig. S1A). Once established, we verified by clonogenic assays that both cell lines displayed significantly increased resistance to irradiation. As shown in Fig. 1A, B, L3^RR^ were significantly more radioresistant (*p* < 0.001) than parental cells, within the 4–6 Gy range; similarly, CMT^RR^ cells showed radioresistance within the 8–10 Gy range (*p* < 0.05) compared to control cells. The radiation inhibitory doses that caused 50% reduction in clonogenicity were as follows: 1.02 Gy for L3 and 3.98 Gy for L3^RR^; 3.67 Gy for CMT and 6.02 Gy for CMT^RR^ (Fig. 1A, B).

Radioresistance was also validated by apoptosis analyses upon irradiation. At 8 Gy, L3^RR^ cells tended to show lower levels of apoptosis than L3 cells, but without reaching statistical significance. However, at higher doses (15 Gy), apoptosis was 50% lower in the RR variant (*p* < 0.01). For CMT and CMT^RR^ cells, apoptosis was reduced by 67.2% and 46.3% in the RR clone when 8 Gy (*p* < 0.05) or 15 Gy (*p* = 0.06) were used, respectively, with compared with parental cells (Fig. 1C). To validate radioresistance in vivo, a fractionated scheme of 3 × 8 Gy was followed for L3 and its RR variant, and 5 × 5 or 5 × 8 Gy for CMT and its RR variant (Fig. 1D, E and Fig. S4A). Tumor volume in L3 tumors treated with this scheme was reduced by ~ 50% compared to untreated controls. However, irradiation following the same protocol did not affect L3^RR^ tumors (Fig. 1D). Similarly, CMT tumors were decreased by 34.9% upon irradiation with 5 × 5 Gy, but CMT^RR^ tumors did not respond to RT (Fig. 1E). Similar results were observed for the scheme 5 × 8 Gy (Fig. S4A).

To better characterize these models, we next studied expression of the DNA damage sensor γH2AX by immunofluorescence, evaluating both signal intensity and number of foci (Fig. 1F-K). When irradiated, signal intensity was significantly increased in both parental and RR cells. However, the signal was significantly higher (*p* < 0.001) in RR cells than in parental cells, suggesting a more efficient activation of DNA repair mechanisms (Fig. 1H, J). Regarding irradiation-induced immunogenic cell death (ICD), both RR cell lines exhibited a significant reduction in the levels of calreticulin exposed on the outer leaflet of the plasma membrane compared with their respective parental counterparts (Fig. 1L, M). Moreover, PD-L1 levels were higher in RR cells when stimulated with IFNγ (Fig. S4B). In addition, functional assays showed that the conditioned media obtained from L3^RR^+RT cells was more able to induce M2-like macrophage polarization and T regulatory cell differentiation than L3 + RT cells (Fig. S4C-D), supporting again the immunosuppressive features of radioresistant cells.

**Fig. 2:**
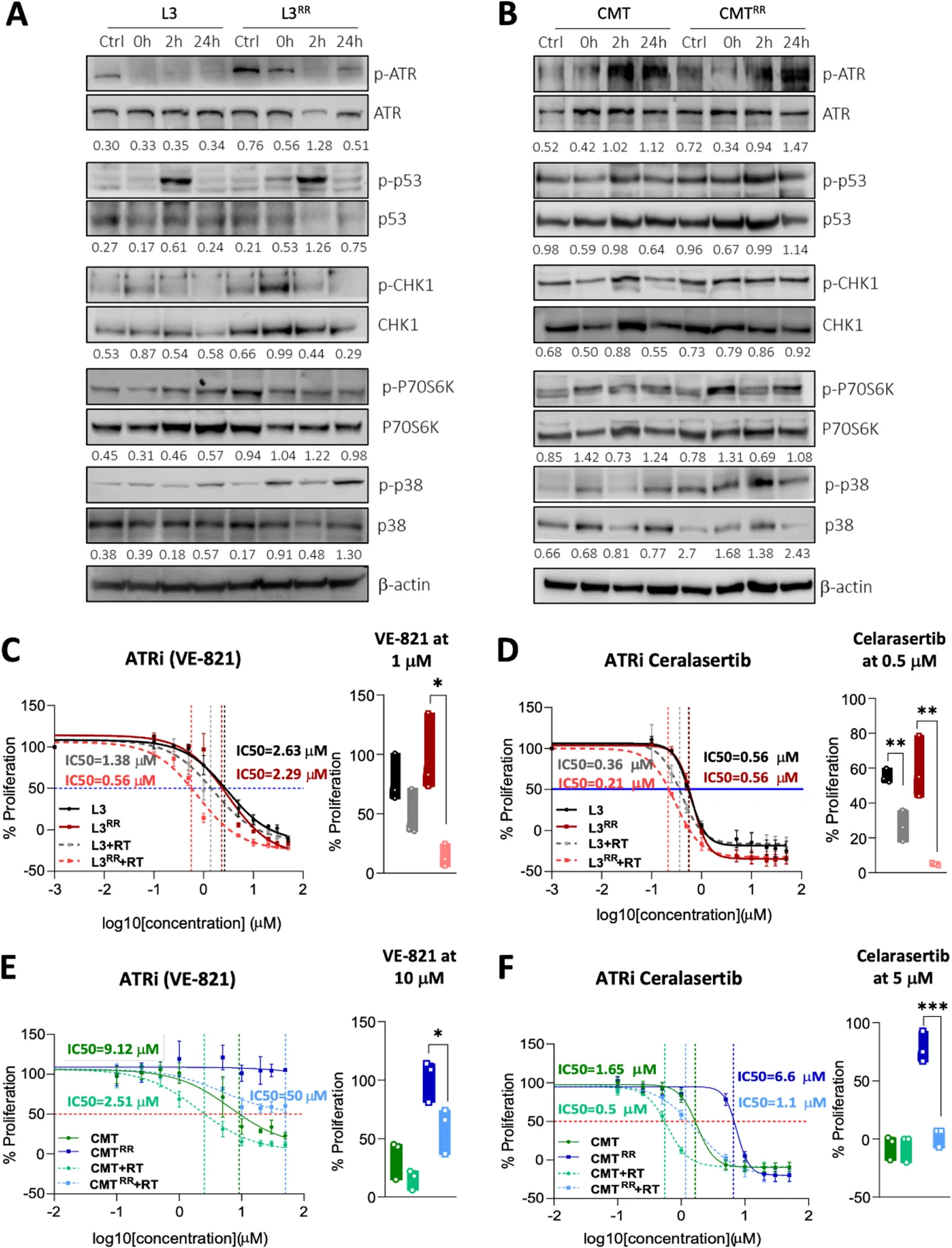
(**A**, **B**) Proteins related with DDR and stress signals, assessed by western blot without irradiation (Ctrl), a few minutes after irradiation (0h), and 2h or 24h after 6 Gy irradiation, in L3, L3^RR^ (**A**), CMT and CMT^RR^ cells (**B**). **C**, **D** Percentage of cell proliferation of L3, L3+RT, L3^RR^ and L3^RR^+RT with or without different concentrations of the ATRi VE-821 or ceralasertib. L3^RR^ cells treated with RT are particularly sensitive to these drugs. **E**, **F** Percentage of cell proliferation of CMT, CMT+RT, CMT^RR^ and CMT^RR^+RT with or without different concentrations of VE-821 or ceralasertib. CMT^RR^ cells are insensitive to VE-821 (IC_50_ not reached) and show low sensitivity to ceralasertib compared to the parental cells. However, both drugs show cytotoxicity in CMT^RR^ cells when combined with RT. Statistical significance was defined as *p*<0.05 (*), *p*<0.01 (**) and *p*<0.001 (***)

Next, proteins of intracellular pathways related to DDR and cellular stress were studied by western blot at 0, 2–24 h post-treatment with 6 Gy irradiation. Basal levels of p-ATR were higher in both RR untreated cells compared with their respective untreated control cells (Fig. 2A, B). Irradiation reduced p-ATR levels in L3 and L3^RR^ cells, whereas a strong increase was observed for CMT and CMT^RR^ cells (Fig. 2A, B). p-P53 levels were induced 2 h after irradiation in both models, but no remarkable differences between RR and parental cells were appreciated. No relevant differences between parental and RR cells or changes upon irradiation were found for p-CHK1. Regarding p-P70S6K, higher levels were seen in L3^RR^ and L3^RR^+RT compared to controls. p-P38 levels were also higher in CMT^RR^, CMT^RR^+RT and L3^RR^+RT compared with their controls (Fig. 2A, B). p-ERK1/2 levels were increased in all cells 2 h after irradiation, with the highest levels observed for both RR cells at this timepoint (Fig. S4E). p-AKT increased after irradiation, but no remarkable differences between parental and RR cells were observed (Fig. S4E).

Based on the general pattern of enhanced activation of p-ATR, p-P70S6K and p-P38 in RR cells, we hypothesized that they would be particularly sensitive to drugs targeting these kinases in combination with RT. The ATR inhibitors (ATRi) VE-821 and ceralasertib (AZD6738) were first tested in L3/L3^RR^ cells (Fig. 2C, D). When no irradiation was applied, IC_50_ for L3 and L3^RR^ cells were similar: 2.63 µM (L3) and 2.9 µM (L3^RR^) for VE-821; 0.56 µM (for both L3 and L3^RR^) in the case of ceralasertib. When cells were irradiated with 6 Gy and exposed to the drugs, IC_50_ were reduced as follows: 1.38 µM for VE-821-treated L3 cells and 0.56 µM for L3^RR^ cells treated with the same drug; 0.36 µM for ceralasertib-treated L3 cells and 0.21 µM for ceralasertib-treated L3^RR^ cells. At a concentration of 1 µM VE-821, a significant reduction in the number of viable cells was only found when RR cells were treated with the drug + RT, but no changes were observed for the parental cells (Fig. 2C). In the case of ceralasertib, at 0.5 µM, significant decrease in viability was found in both L3 + RT and L3^RR^ +RT (*p* < 0.01), but the magnitude of the effect was much more pronounced in the RR clone (91.5 vs. 51.7%) (Fig. 2D). Ceralasertib was more effective at inducing apoptosis in RR + RT cells than in parental cells (treated or no with RT) (Fig. S4F).

In the absence of irradiation, CMT^RR^ cells demonstrated insensitivity to VE-821 (IC_50_ not reached) and low sensitivity to ceralasertib (IC_50_ 6.6 µM), compared to CMT parental cells (IC_50_ 9.12 µM for VE-821 and IC_50_ 1.65 µM for ceralasertib) (Fig. 2E, F). However, as for L3^RR^ cells, VE-821 or ceralasertib in combination with RT were particularly effective in CMT^RR^ cells (Fig. 2E, F).

Experiments using the P70S6K inhibitor (PF-4708671) and the P38 inhibitor (Ralimetinib Dimesylate) showed no remarkable differences in RT sensitivity between parental vs. RR cells (Fig. S4G-J). Taken together, these results show that combination between ATR inhibitors and RT can be therapeutically effective against cancer cells with acquired radioresistance.

**Fig. 3:**
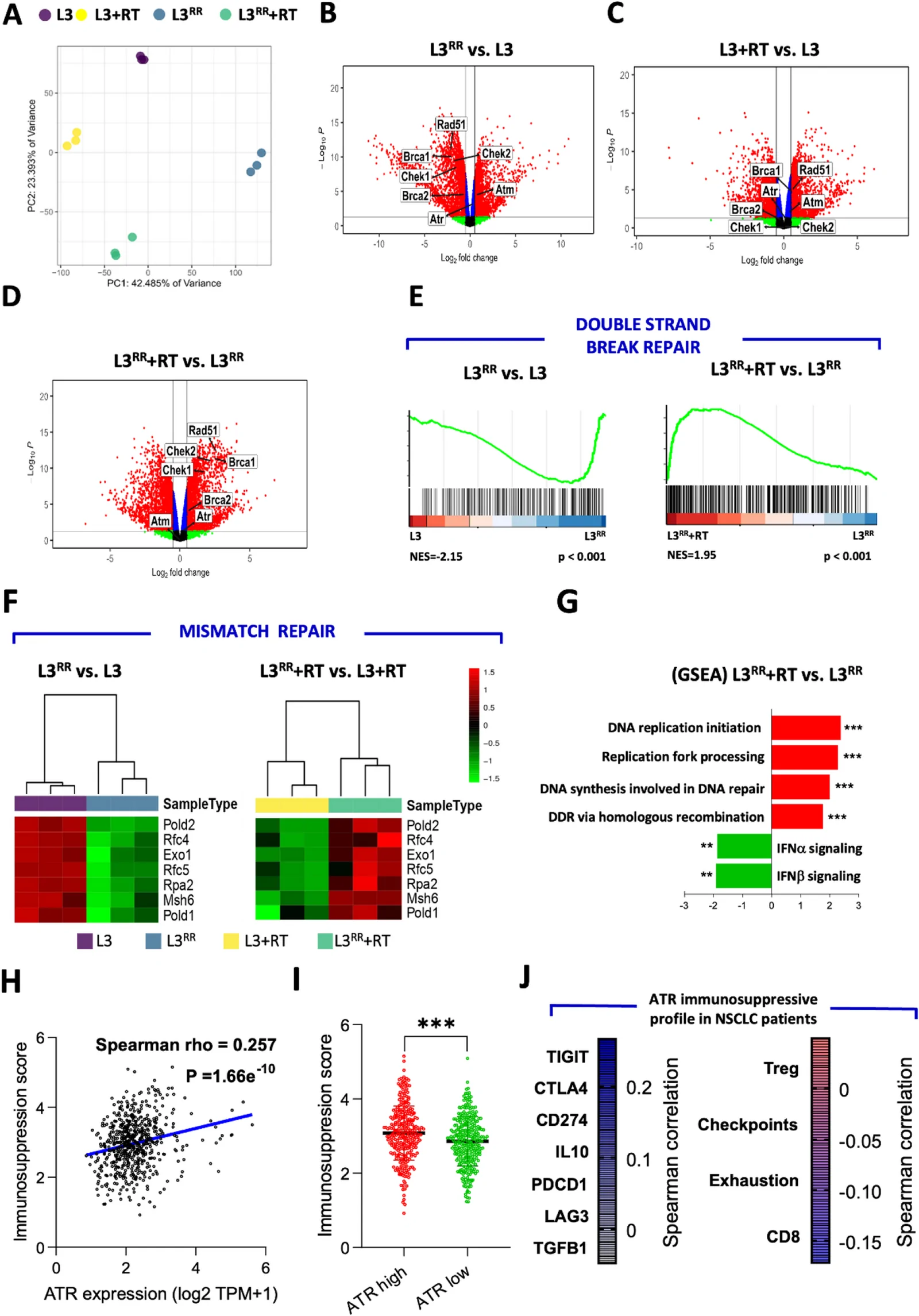
(**A**) PCA shows diversity in the transcriptomic profiles of L3, L3+RT, L3^RR^ and L3^RR^+RT cells. **B**-**D** Volcano plots to graphically show significant changes in gene expression between L3^RR^ and L3 (**B**), L3+RT and L3 (**C**), and L3^RR^+RT and L3^RR^ (**D**). The red color represents genes whose log2FC <-1 and p-value <0.05 or log2FC >1 and p-value <0.05. The blue color represents genes whose log2FC >-1 and p-value <0.05 or log2FC <1 and p-value <0.05. The green color represents genes whose log2FC <-1 and p-value >0.05 or log2FC >1 and p-value >0.05. Some relevant genes related to DDR are highlighted. **E** GSEA analysis of the category “Double strand break repair” shows significant negative enrichment (normalized enrichment score, NES: -2.15) in L3^RR^ compared to L3 cells (p<0.001). On the contrary, significant positive enrichment is found (NES: 1.95, p<0.001) in L3^RR^+RT compared with L3^RR^ cells, suggesting effective activation of DDR pathways upon radiotherapy treatment. **F** A similar pattern is observed with the category “Mismatch repair” using heatmap representation, where red and green indicate increased and decreased relative expression, respectively, across samples. **G** GSEA analysis resulting from the comparison between L3^RR^+RT and L3^RR^ cells. Red color indicates significant positive enrichment of categories involved in DNA replication, repair and homologous recombination. Significant negative enrichment (green) in type-I interferons suggests downregulation of immunostimulatory signals. **H** Correlation between ATR mRNA levels and an immunosuppressive score, in LUAD patients (n=600). Data were retrieved from The Cancer Genome Atlas (TCGA) database. The score includes expression of the following genes: PD-L1, PDCD1 (PD-1), CTLA4, LAG3, TIGIT, TGFB1 and IL10. **I** High mRNA levels of ATR (over the median) are associated with high immunosuppressive score. **J** Correlation coefficients of immune-related genes and immune cell populations (inferred by CIBERSORT) and ATR expression, in NSCLC patients. Statistical significance was defined as *p*<0.05 (*), *p*<0.01 (**) and *p*<0.001 (***)

To investigate gene expression profiles associated with the acquisition of radioresistance, we compared the transcriptome of L3^RR^ and parental L3 cells under irradiated and non-irradiated conditions. Principal component analyses (PCA) showed that gene expression profiles of L3, L3 + RT, L3^RR^ and L3^RR^+RT were substantially different (Fig. 3A). In the absence of irradiation, 3,156 genes were downregulated and 1,863 genes were upregulated in L3^RR^, as compared to parental cells. Some of the downregulated genes in L3^RR^ cells were related to DDR, such as *Rad51*, *Brca1*, *Brca2*, *Chk1* and *Chk2*, whereas an increase in *Atm* and *Atr* was also observed (Fig. 3B). Gene Ontology (GO) analyses revealed several categories significantly altered in the RR cell line, which can be summarized in the following main classes: “DNA replication”, “cell cycle” and “DNA repair” (Fig. S5A). Strikingly, Gene Set Enrichment Analysis (GSEA) analyses revealed significant negative normalized enrichment score (NES) in 19 gene sets related with these processes (Table S6).

The extent of DNA damage was also studied through whole exome sequencing and estimation of tumor mutational burden (TMB) by quantifying the average number of SNP (single nucleotide polymorphism) per megabase (Mb). While TMB for L3 was 17.15 SNP/Mb, that corresponding to L3^RR^ cells was 23.13 SNP/Mb (~ 24% increase; Fig. S5B). Although both L3^RR^ and CMT^RR^ cells tended to proliferate less than their corresponding parental cells, no statistical differences were found (Fig. S5C, D).

The effect of irradiation (6 Gy) on L3 and L3^RR^ transcriptomes was next investigated. When L3 cells were irradiated, expression of 3,160 genes was found to be differentially altered (1,050 upregulated and 2,110 downregulated) (Fig. 3C). Irradiation of its RR counterpart with the same dose caused significant changes in 5,468 genes (2,141 upregulated and 3,227 downregulated) (Fig. 3D). We focused on gene expression changes in DDR-related genes upon irradiation in both cell lines. Interestingly, DDR genes were significantly increased in irradiated L3^RR^ cells compared with irradiated L3 cells. Examples of upregulated genes involved in double stranded DNA break repair were *Chk1*, *Chk2*, *Rad51* and *Brca1* (*p* < 0.001 in all cases) (Fig. 3D). Consistent with these findings, GSEA showed positive enrichment of the “double strand break repair” category in L3^RR^+RT condition (Fig. 3E). The same pattern was observed for the category “mismatch repair”, with increased expression of genes such as *Pold1*, *Pold2*, *Exo1*, *Rpa2* and *Msh6* in L3^RR^ cells treated with RT (Fig. 3F). Of note, the features described for RR cells were maintained over the course of the experiments, without the need of giving maintenance irradiation doses to perpetuate the phenotype, thus showing permanent adaptation. Therefore, although untreated RR cells appear to show chronic tolerance to DNA damage, with downregulation of DDR pathways, these cells are more proficient at DNA repair than their isogenic counterparts when challenged with irradiation.

In RT-treated L3^RR^ cells, signaling through type I interferons were significantly downregulated compared with its parental counterpart, suggesting decreased immunogenicity of these cells (Fig. 3G). Gene categories “DNA replication” and “Cell cycle checkpoint” were also positively enriched in L3^RR^+RT compared to L3^RR^ cells (Fig. S5E), which suggests the induction of positive signals for survival and proliferation under the stress of RT.

We also wondered whether ATR could be contributing to the immunosuppressive phenotype observed in our RR cancer cell models. In tumor samples from NSCLC untreated patients, ATR mRNA levels were significantly correlated (*p* < 0.001) with an immunosuppression score that included genes *PD-L1*, *PDCD1 (PD-1)*, *CTLA4*, *LAG3*, *TIGIT*, *TGFB1* and *IL10* (Fig. 3H). Classification of NSCLC patients based on the median ATR tumor expression showed that those with “high levels” were significantly associated with immunosuppression (Fig. 3I). In addition, through deconvolution analyses, we also found that ATR expression associated with expression of genes that define T-regulatory cell populations and immune populations expressing immune checkpoints (see Fig. 3J).

To study further if ATR could regulate immunomodulatory pathways (more specifically type I IFN) in cancer cells, L3 and L3^RR^ cells were treated with the ATRi ceralasertib, RT or a combination of both (Fig. S6A). In L3 cells, p-ATR levels were reduced, as expected, by the ATRi-containing regimes. While cGAS levels were unchanged, p-STING and total TBK1 levels were increased in ATRi and ATRi + RT conditions. Also as expected, γH2AX was strongly induced in both ATRi- and ATRi + RT-treated cells. These results suggest that ATR blockade may stimulate IFN-I-STING-related signaling in parental cells. In RR cells, as p-ATR were much more abundant than in parental cells, the ATRi only inhibited its levels partially. γH2AX was induced more than in the parental cells, again supporting their higher DNA damage response. The IFN-I-STING-related proteins followed a different modulation pattern compared to the L3 cells, with less clear induction of expression, suggesting once again lower immune stimulation.

### Essential role of the immune system in maintaining radioresistance in vivo

**Fig. 4:**
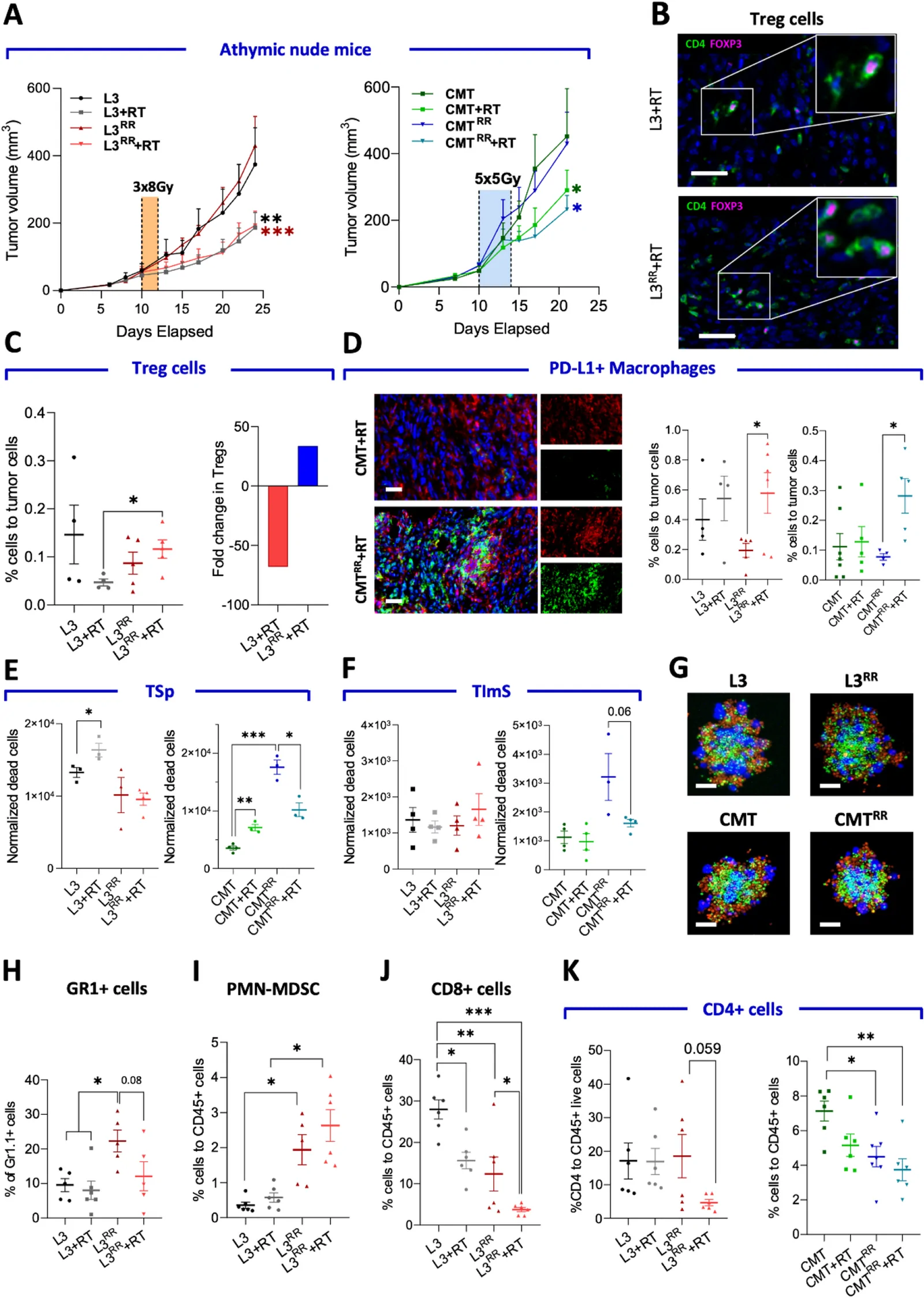
(**A**) Radiotherapy experiments performed in athymic nude mice bearing L3, L3^RR^, CMT, and CMT^RR^ tumors. Both RR models remained responsive to their corresponding RT regimens, suggesting a contribution of the immune system to the acquisition and maintenance of radioresistance. **B** Representative multiplex immunofluorescence (mIF) images showing colocalization of CD4^+^ and FOXP3^+^ cells in L3 and L3^RR^ tumors following radiotherapy (RT). Scale bar: 30 µm **C**. Quantification of T regulatory cells (Treg cells: CD4^+^FOXP3^+^), expressed as percentage and fold change, comparing L3+RT vs. L3 tumors and L3^RR^+RT vs. L3^RR^ tumors. **D** Representative mIF images and their corresponding quantification demonstrating a significant increase in the percentage of PD-L1^+^ macrophages in both irradiated L3^RR^ and CMT^RR^ models. Scale bar: 20 µm. F4/80^+^ cells are shown in red color, PD-L1^+^ cells in green; DAPI (blue) was used to stain the nuclei. **E**,** F** Quantification of dead cells per mold present in TSp (**E**) and TImS (**F**) formed by L3, L3^RR^, CMT and CMT^RR^ cells with or without irradiation. **G** Representative images showing TImS formed by L3, L3^RR^, CMT and CMT^RR^ cells at day 0. Scale bar 100 µm. M2-like macrophages were tracked in red color, T regulatory cells in green; DAPI (blue) was used to stain the nuclei. **H** Analysis of GR1^+^ cells evaluated by immunohistochemistry, in L3, L3^RR^, L3+RT and L3^RR^+RT tumors. **I** Flow cytometry analysis showing increase (*p*<0.05 for L3^RR^ and L3^RR^^+^RT) in the percentage of PMN-MDSC cells (CD45^+^CD11b^+^Ly6G^high^Ly6C^low^). **J** Flow cytometry analysis showing a significant reduction in the percentage of CD8^+^ cells in irradiated L3^RR^ tumors. **K** Flow cytometry analysis showing a decrease in the percentage of CD4^+^ cells (*p*<0.01 in CMT^RR^+RT) in both RR models following RT. Statistical significance was defined as *p*<0.05 (*), *p*<0.01 (**) and *p*<0.001 (***)

To evaluate the contribution of the immune system to radioresistance in vivo, we applied the corresponding treatment schedules to athymic immunodeficient nude mice (Fig. 4A). Strikingly, tumors from both L3^RR^ and CMT^RR^ groups responded to RT similarly to those of radiosensitive models. This implies that the immune system is playing a predominant role in radioresistance in vivo.

In subsequent experiments performed in immunocompetent mice, we focused on the identification of potential immune populations and cytokines/chemokines involved in radioresistance and gene expression pathways that may regulate this phenotype. First, the number of immunosuppressive Treg cells and M2-like macrophages were evaluated by multiplex immunofluorescence (mIF) in tumors from L3-derived models. While the number of tumor infiltrating Treg cells was decreased in L3 + RT compared to controls, the opposite pattern was observed for L3^RR^+RT tumors (Fig. 4B, C). A statistically significant increase in Treg cells was found in L3^RR^+RT tumors compared to L3 + RT tumors (Fig. 4C). The immunosuppressive population of PD-L1^+^ macrophages [28] was significantly increased in both irradiated RR models, while it was unchanged in radiosensitive tumors (Fig. 4D).

We hypothesized that these immunosuppressive cell populations could protect cancer cells from RT-induced cell death. To test this experimentally, we established 3D co-cultures containing tumor cells, Treg cells and M2-like macrophages (TImS) using cell trackers, as previously described by our group [15]. The control arm was the tumor spheroid (TSp) without immune cells (see experimental scheme in Fig. S6B). In TSp, RT significantly increased the number of dead L3 (*p* < 0.05) and CMT (*p* < 0.01) cancer cells (Fig. 4E). While non-irradiated L3^RR^ cells showed fewer dead cells within the TSp than non-irradiated parental cells, the opposite result was observed for CMT^RR^ cells (Fig. 4E). This is in keeping with the fast growth of CMT^RR^ cells in spheroids, which leaves a necrotic core in the middle [15]. Unlike for L3 parental cells, administration of RT to L3^RR^ cells did not change the number of dead cells in the TSp. Surprisingly, a significant reduction in the number of dead cells was observed in irradiated CMT^RR^ TSp when compared to their respective untreated TSp (Fig. 4E). The likely explanation is that RT in this model impairs cell proliferation rather than killing cancer cells, which may lead to a reduced necrotic core in the TSp. In TImS from L3 and L3^RR^ cells, RT did not cause apoptosis of tumor cells, demonstrating the protective role of these immunosuppressive populations on cancer cell death upon irradiation. Similar results were found for RT-treated CMT-derived TImS (Fig. 4F). Again, a partial reduction in the number of dead cells was observed in CMT^RR^ cells when RT was applied (*p* = 0.06 compared to untreated CMT TImS), which may also have an explanation in the reduction of the necrotic core (Fig. 4F). Representative images of the TImS are shown in Fig. 4G and Fig. S6C. Taken together, these results indicate that immunosuppressive M2-like macrophages and Treg cells protect RR cancer cells from irradiation-mediated cytotoxicity. Other studies have also described the role that macrophages can play in protecting irradiated cancer cells from dying, but mechanisms are still elusive [29, 30]. Macrophages are particularly resistant to radiotherapy [29]. Xu et al., [30] suggested that protection was due to secreted factors (such as TNF-α/VEGF). A more recent study has shown a paracrine/bystander cytoprotective effect elicited by the macrophages, without defining the mechanisms [31]. In our models, conditioned media from irradiated M2-like macrophages did not protect cancer cells from irradiation-induced apoptosis (not shown). CCL2 was highly upregulated in irradiated macrophages (Fig. S6D), but adding recombinant CCL2 to our irradiated cancer cells did not convey protection either (not shown), despite reports suggesting otherwise in pancreatic cancer cells expressing CCR2 [32]. Therefore, in our models, direct cell-to-cell (cancer cell-macrophage) signaling could play a critical role in this shielding effect, which will be addressed in future studies.

Next, we assessed in more detail immune infiltrates in RR tumors treated or not with RT, using flow cytometry, mIF and immunohistochemistry. The GR1^+^ (Ly6G^+^/Ly6C^+^) population, strongly expressed in monocytic (M) MDSC and polymorphonuclear (PMN) MDSC, inflammatory monocytes and neutrophils (although in a lesser extent), was increased in untreated L3^RR^ tumors compared to L3 controls, with a reduction upon exposure to RT (Fig. 4H and Fig. S7A). In L3^RR^ and L3^RR^+RT groups, PMN-MDSC were significantly higher than in controls (Fig. 4I), and the same trend was found for CMT^RR^ and CMT^RR^+RT tumors (Fig. S7B). Flow cytometry showed that Treg cells tended to be higher in both groups of RR tumors treated with RT (Fig. S7C). The number of tumor infiltrating CD8^+^ lymphocytes, PD-1^+^ cells and CD8^+^PD-1^+^ was also evaluated, showing in all cases a significant reduction in L3^RR^ tumors compared to controls, particularly after administration of RT (Fig. 4J and Fig. S7D-G). The same trend was observed for CMT^RR^ and CMT^RR^+RT tumors (Fig. S7H). The number of CD4^+^ lymphocytes tended also to be lower in L3^RR^+RT compared to the other groups. Such reduction was statistically confirmed in the CMT^RR^+RT model (Fig. 4K). Considered jointly, these results confirmed the immunosuppressive phenotype of RR tumors.

**Fig. 5:**
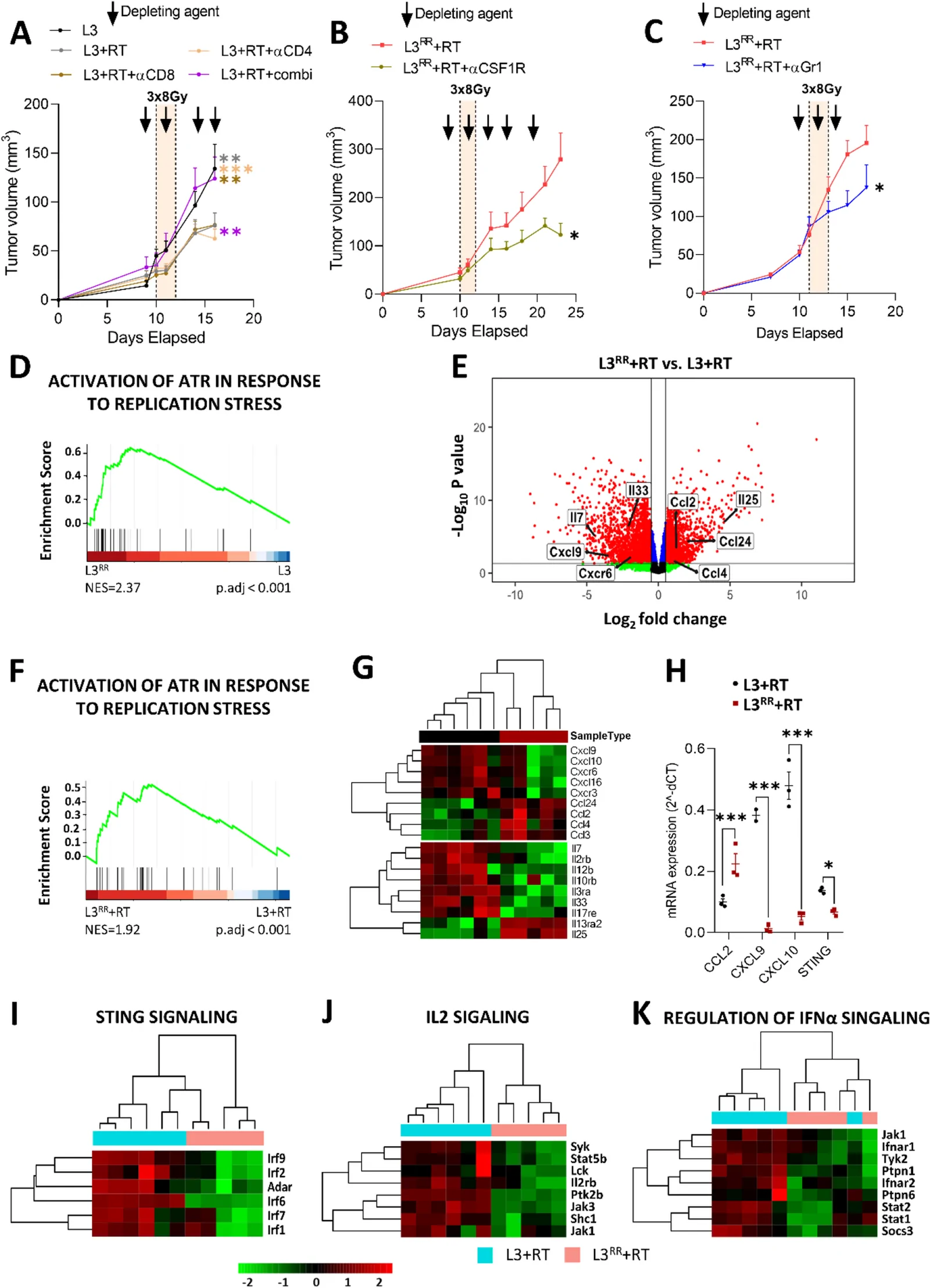
(**A**) Depletion experiment in the L3 model to study the effect of CD8^+^ and CD4^+^ immune cell populations in the response to RT (3x8Gy). Blockade of both CD4^+^ and CD8^+^ T cells completely eliminated the effect of RT. **B** Depletion experiment in the L3^RR^ model to study the involvement of CSF1R^+^ cells in the acquisition of radioresistance. **C** Depletion experiment in the L3^RR^ model to study the involvement of GR1^+^ cells in the acquisition of radioresistance. **D** GSEA analysis of the category “Activation of ATR in response to replication stress” shows significant positive NES in L3^RR^ tumors compared to parental tumors (NES: 2.37; *p*<0.001). **E** Volcano plot showing differential expression of several cytokines and interleukins related to immune system trafficking in L3^RR^+RT tumors compared to L3+RT tumors. The red color represents genes whose log2FC <-1 and *p*-value <0.05 or log2FC >1 and p-value <0.05. The blue color represents genes whose log2FC >-1 and *p*-value <0.05 or log2FC <1 and *p*-value <0.05. The green color represents genes whose log2FC <-1 and p-value >0.05 or log2FC >1 and *p*-value >0.05. **F** GSEA analysis of the category “Activation of ATR in response to replication stress” shows significant positive NES in L3^RR^+RT vs. L3+RT tumors (NES: 1.92; *p*<0.001). **G** Heatmap representation of some of the most relevant cytokines and interleukins differentially expressed in L3^RR^+RT vs. L3+RT tumors. **H** mRNA expression levels to validate the significant increase of CCL2 and decreased levels of CXCL9, CXCL10 and STING in L3^RR^+RT tumors compared to L3+RT tumors. **I**-**K**. Heatmap representation of “STING signaling” (**I**), “IL2 signaling” (**J**) and “Regulation of IFNα signaling” (**K**) showing decreased expression (green) of key genes belonging to these pathways in L3^RR^+RT tumors, as compared to L3+RT tumors. Statistical significance was defined as *p*<0.05 (*), *p*<0.01 (**) and *p*<0.001 (***)

To assess whether the immune populations were playing a role in the response or resistance to RT, we performed depletion experiments. CD4^+^ and/or CD8^+^ lymphocytes were depleted to test if the response to RT in L3 parental tumors would be impaired. Strikingly, only the combined depletion of CD4^+^ and CD8^+^ was able to completely abolish the effect of RT, while the depletion of single populations had no impact on the response (Fig. 5A). This suggests a cooperative role of both types of lymphocytes to mediate an effective RT response. Based on our results showing enhanced tumor infiltration of immunosuppressive macrophages and PMN-MDSC in L3^RR^ tumors treated with RT, we hypothesized that depletion of the CSF1R^+^ or GR1^+^ populations would cause radiosensitization. As shown in Fig. 5B, C, L3^RR^ tumors were significantly radiosensitized in the presence of these depleting antibodies, with 56% reduction in tumor volume for αCSF1R and 30% for αGR1.

RNAseq was performed on RT-treated or untreated L3 and L3^RR^ tumors grown in immunocompetent mice. PCA showed that the transcriptome of untreated L3-derived tumors (+/- RT) was markedly different to that of L3^RR^-derived tumors (Fig. S7I), suggesting that RR tumors differed significantly from their respective radiosensitive controls. Gene expression analysis revealed 3,278 upregulated and 6,901 downregulated genes in non-irradiated L3^RR^ tumors with respect to non-irradiated control tumors (Fig. S7I). Unlike for in vitro findings in L3^RR^ cells, DDR genes and GSEA categories related to DNA repair were upregulated in L3^RR^ tumors compared to their corresponding controls (with increased expression of genes such as *Rfc4*, *Exo1*, *Rad51b*; Fig. S7I and Table S7), which may imply context-dependent gene expression changes depending on external stressful conditions. Particularly relevant was the significant enrichment of “Activation of ATR in response to replication stress” (NES: 2.37, *p* < 0.0001) (Fig. 5D). The higher induction of DDR pathways in vivo in RR tumors relative to controls, may be explained by their increased TMB, which could impose a selective pressure by immune surveillance.

Inhibition of the innate and adaptive immune system was very prominent in non-irradiated L3^RR^ tumors, as shown by GSEA and heatmap analyses. Significant negative enrichment of “IFNγ signaling” (NES: -2.28, *p* < 0.001), “IFN-responsive genes” (-2.14, *p* < 0.0001) and “Innate immune sensing” (NES: -1.66, *p* < 0.0001) was observed (Table S7). Some of the downregulated genes were related to innate immunity, such as cGAS, which activates STING to induce IFN-dependent responses. Therefore, transcription patterns of non-irradiated RR tumors were associated with enhanced DDR and immunosuppression.

To study whether ATR could also be participating in the immunosuppressive traits of L3^RR^ tumors, we treated mice with ceralasertib in monotherapy or combined with RT. Ceralasertib alone caused a very modest antitumor effect in these tumors, but the therapeutic effect combined with RT was highly improved (*p* < 0.01 compared to ceralasertib alone; *p* < 0.001 compared to RT alone or vehicle-treated control mice; ~52% tumor growth inhibition compared to controls (Fig. S8A)). Quantification of intratumor levels of CD8^+^, CD11C^+^, FOXP3^+^ and F4/80^+^ cells by immunohistochemistry in the ceralasertib group, gave the following results when compared to the control group: While the number of CD8^+^ T lymphocytes decreased in treated animals (Fig. S8B), as previously reported for these rapidly dividing cells [33], the number of F4/80^+^ cells increased (*p* = 0.06) (Fig. S8B). Infiltration of FOXP3^+^ and CD11C^+^ cells did not differ between groups (not shown). Moreover, in in vitro experiments, the ATRi induced the expression of the immune chemoattractant chemokines CXCL9 and CCL2 in L3^RR^ cells (Fig. S8C). This effect was also found for L3 cells, but the induction was much less profound (Fig. S8C).

Next, we studied transcriptomic changes in L3 and L3^RR^ tumors after irradiation. Differential expression analyses showed 5,992 downregulated and 2,536 upregulated genes in L3^RR^+RT tumors compared to L3 + RT tumors (Fig. 5E). Again, transcriptomic patterns indicated upregulation of DDR genes; it is worth stressing an enrichment of the category “Activation of ATR in response to replication stress” (NES:1.92, *p* < 0.0001) (Fig. 5F) and downregulation of genes involved in immune system activation (Table S8).

Functional assays were also performed to test if conditioned media from L3 + RT/L3^RR^+RT cells exposed to the ATRi would affect Treg cell differentiation or M2-macrophage polarization (Fig. S8D, E). Ceralasertib reduced Treg cell differentiation (*p* = 0.08) in conditioned media from L3^RR^+RT cells; also, M2 polarization (*p* < 0.01) in conditioned media from L3 + RT cells, with a non-significant trend in media from L3^RR^+RT cells (*p* = 0.2). It is possible that the ATRi is insufficient to eliminate the strong immunosuppressive phenotype of RR cells to block M2 polarization. Nonetheless, taken together, all these results show that blockade of the ATR signaling may reduce immunosuppression in irradiated RR tumors/cells.

The RNAseq data from tumors also showed a significant negative enrichment of cGAS/STING, IL2, IL15 and NK signaling in L3^RR^+RT tumors. Consistent with these findings, the following chemokines/cytokines were upregulated: CCL2, chemoattractant for macrophages; CCL4, which recruits M2-like macrophages and Treg cells; and IL25, known to recruit immunosuppressive neutrophils through IL8. On the contrary, a remarkable downregulation in the expression of proinflammatory regulators (i.e. IL33, IL7, CXCL9, CXCL10, CXCR6 and CXCR3), mostly involved in the recruitment of different cytotoxic immune populations such as NK or T cells, could contribute to the immunosuppressive TME of these tumors (Fig. 5G). Some of the genes with potential therapeutic implications in our study were validated by qPCR in L3^RR^+RT and CMT^RR^+RT models. CCL2 levels were strongly upregulated (*p* < 0.001), whereas CXCL9 (*p* < 0.001), CXCL10 (*p* < 0.001) and STING (*p* < 0.05) were downregulated in L3^RR^+RT tumors, compared with L3+RT tumors (Fig. 5H). The same trend (except for STING) was found in CMT^RR^+RT tumors (Fig. S8F). Genes of the IRF family, JAK/STAT and IFNα signaling pathways were also downregulated in irradiated L3^RR^ tumors compared to their controls (Fig. 5I-K).

We were particularly interested in the abrogation of CXCL9 in the RR tumors treated with RT, because of its potential therapeutic implications. To ascertain the functional relevance of such finding, CXCL9 was overexpressed in L3^RR^ cells (which we named L3^RR^-CXCL9 OE) (Fig. S8G). The conditioned media from these cells was more proficient at attracting splenocytes (*p* < 0.001) than the scramble (Scr)-infected controls (Fig. S8H). When injected in syngeneic mice, CXCL9-overexpressing cells grew at a similar pace compared with controls (not shown). However, these tumors showed a tendency to be more infiltrated by immune cells (CD45^+^, *p* = 0.05; CD11b^+^, *p* = 0.1) and less by immunosuppressive PMN-MDSC (*p* = 0.07) than controls (Fig. S8I-K). No significant differences or trends were observed for other immune populations, including lymphocytes, NKs or macrophages (not shown).

### Co-targeting the immunosuppressed TME and ATR reverts radiotherapy insensitivity and reduces tumor growth

**Fig. 6:**
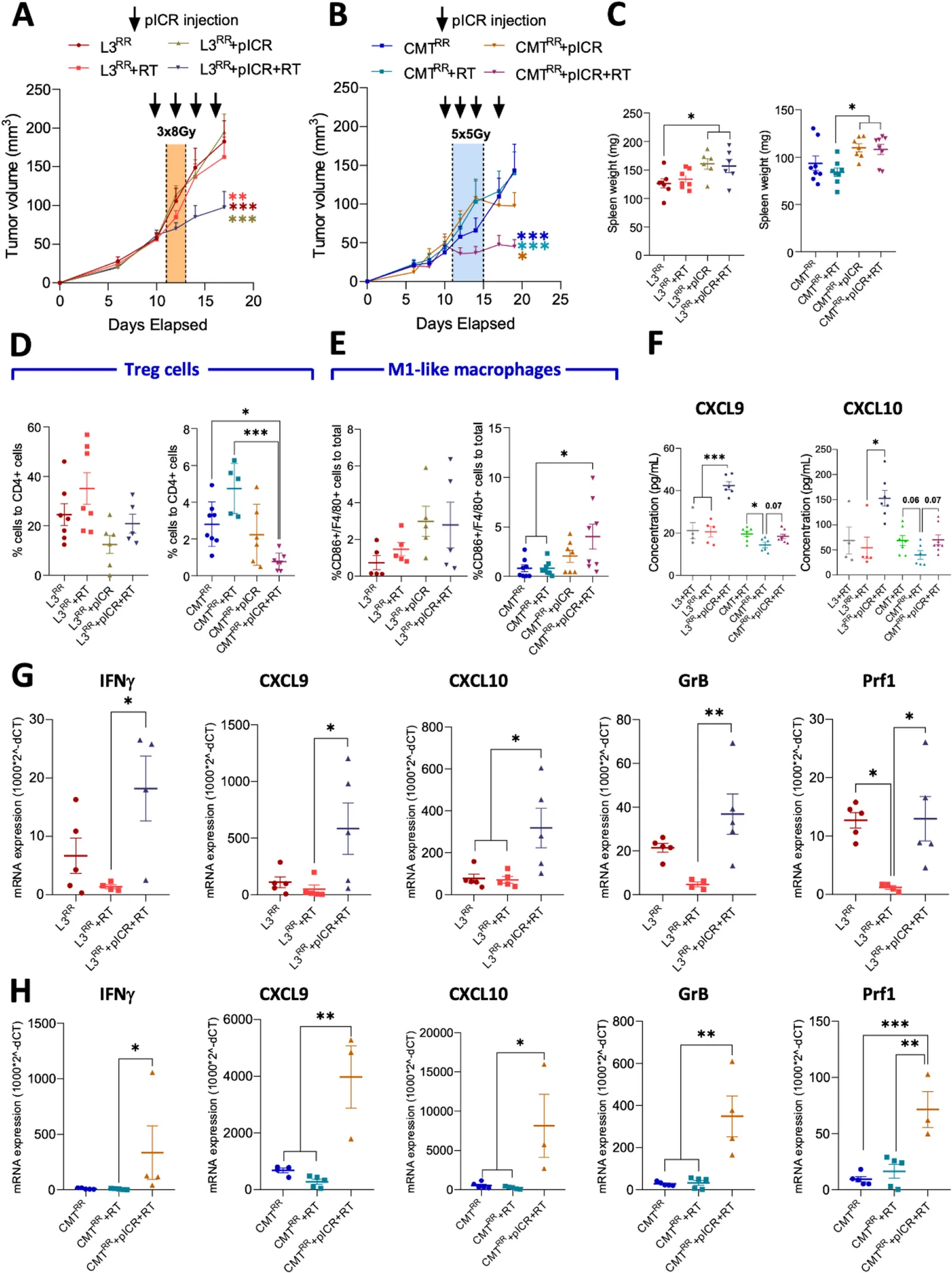
(**A**, **B**) Tumor growth of L3^RR^ (**A**) and CMT^RR^ (**B**) mice treated with poly(I:C)+R848 (pICR) (25 μg each, intratumor, i.t.) and/or RT. **C** Spleen weight of mice at the end of the experiment. A significant increase is observed in all the groups treated with pICR alone or in combination with RT. **D** Analyses of infiltrated Treg cells show a decrease in both RR tumors upon combination of RT and pICR (*p*<0.001 for CMT^RR^+RT+pICR compared to CMT^RR^+RT). **E** Analyses of M1-like macrophages (CD86^+^F4/80^+^) show an increase in both RR tumors upon combination of RT and pICR (*p*<0.05 for CMT^RR^+RT+pICR compared to CMT^RR^ or CMT^RR^+RT). **F** Plasma levels of CXCL9 and CXCL10. Both cytokines show an increase in L3^RR^ (*p*<0.001 and *p*<0.05) and CMT^RR^ (*p*=0.07) tumors treated with RT+pICR. **G, H** mRNA expression levels of different immunomodulatory markers. Analysis show that combination of RT+pICR significantly increases the levels of IFNγ, CXCL9, CXCL10, granzyme B (GrB) and perforin (Prf1) in L3^RR^(**G**) and CMT^RR^ tumors (**H**). Statistical significance was defined as *p*<0.05 (*), *p*<0.01 (**) and *p*<0.001 (***)

We evaluated strategies to radiosensitize RR tumors by a strategy involving direct targeting of cancer cells with the ATRi (with an indirect effect on the TME) and by activating the innate immune system. Based on prior work from our group [13], intratumoral administration of a combination of poly(I:C) (pIC) and R848, agonists of TLR3 and TLR7/8, respectively, was tested. This treatment induces M1-like macrophage repolarization, reduces Treg cell infiltration, increases CD4^+^ and CD8^+^ T cell infiltration and promotes activation of the cGAS–STING pathway through TLR signaling [13]. Combined treatment with pIC+R848 (pICR) and RT resulted in a highly significant reduction in tumor volume compared with RT alone in both L3^RR^ (~ 50%) and CMT^RR^ (~ 65%) models (Fig. 6A, B). Administration of pICR alone did not affect tumor growth in the L3^RR^ model, whereas a modest reduction was observed in CMT^RR^-bearing mice. Analysis of splenic weight revealed a significant increase in spleen size, in mice treated with pICR alone or in combination with RT (Fig. 6C), which could be consistent with systemic immune activation. No signs of toxicity were observed during the course of the experiment, and body weight remained stable across all treatment groups (Fig. S9A, B).

To further characterize changes in the TME, mIF analysis was performed in treated and untreated RR models. A non-significant increase in the proportion of Treg cells was observed in both RR models following RT alone (Fig. 6D). In contrast, pICR + RT resulted in a significant drop of Treg cell proportion in the CMT^RR^ model, with a similar trend observed in L3^RR^ tumors (Fig. 6D). Analysis of F4/80^+^ cells revealed an increase in total infiltrating macrophages, in both RR models (*p* < 0.05 in CMT^RR^) treated with pICR + RT (Fig. S9C). A trend toward an increased proportion of CD86⁺F4/80⁺ (M1-like) macrophages was observed in L3^RR^ tumors treated with pICR, either alone or in combination with RT. A similar increase was also detected in L3^RR^+RT tumors, which was statistically significant (*p* < 0.05) compared to both RT-treated and untreated control groups (Fig. 6E).

A multiplex cytokine analysis focused on immune mediators associated with cytotoxic lymphocyte recruitment and macrophage polarization was then performed. Plasma levels of CXCL9 and CXCL10, chemokines implicated in the recruitment of cytotoxic T cells and NK cells, as well as in M1 macrophage polarization [34], were significantly increased in mice treated with pICR + RT compared with RT alone in the L3^RR^ model (Fig. 6F), although such pattern was not observed for the CMT^RR^ model (Fig. 6F). No significant changes were detected in plasma levels of IL1β, CXCL16, IL25, CCL2 and IL6 (Fig. S9D). We also assessed the expression of CXCL9 and CXCL10, together with cytotoxic activation markers including IFNγ, granzyme-B (GrB) and perforin (Prf1), within the tumor compartment by qPCR. A highly significant upregulation of all markers in both L3^RR^ and CMT^RR^ tumors treated with pICR + RT, compared with RT alone, was obtained (Fig. 6G, H).

**Fig. 7:**
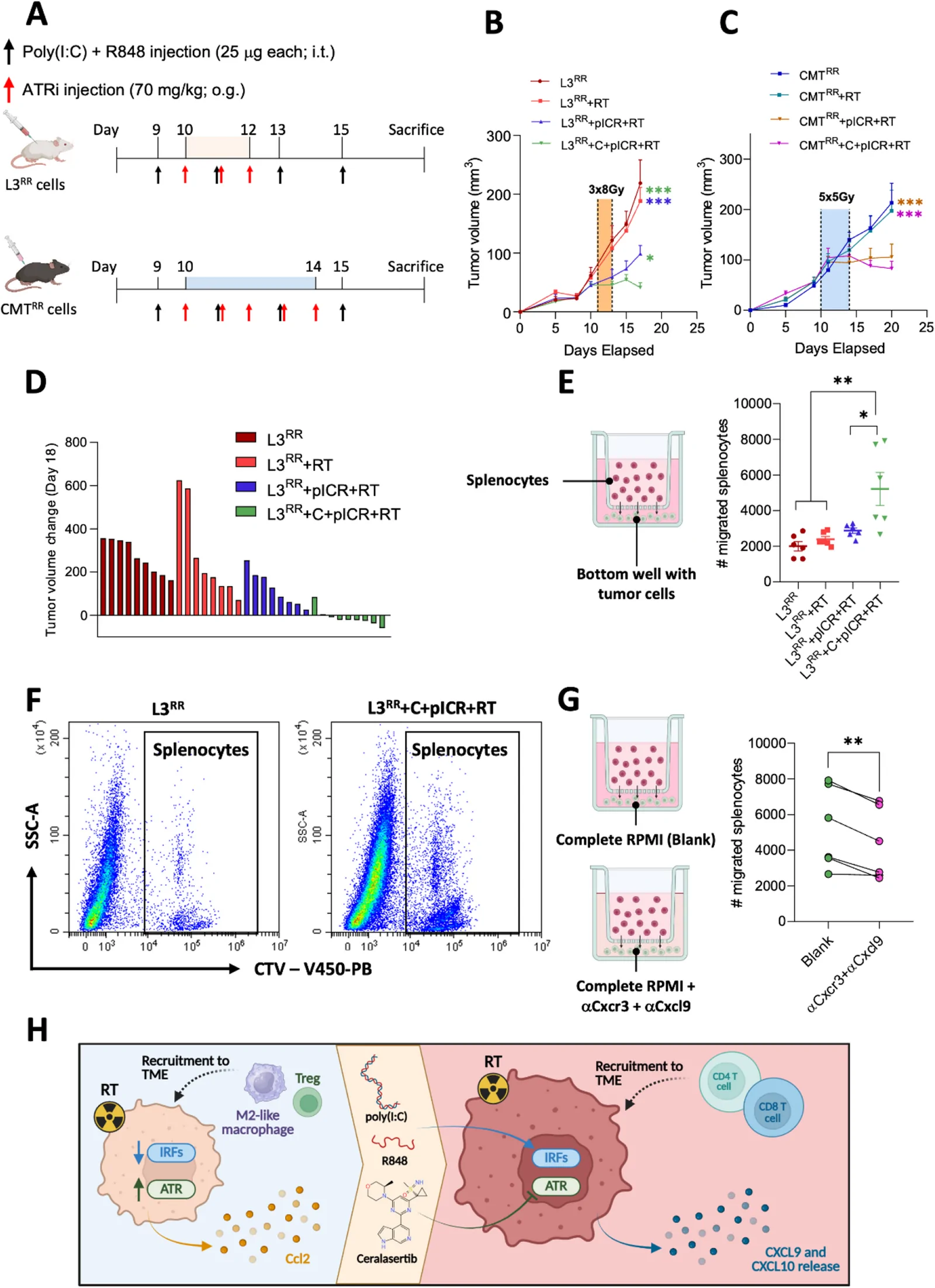
(**A**) Diagram showing different treatments in L3^RR^ and CMT^RR^ models. In both cases, poly(I:C) and R848 were intratumorally (i.t.) administered (25 μg each) every 48h, starting one day prior to the corresponding RT schedules. In the case of ceralasertib, it was administered by oral gavage (o.g.) (70 mg/kg) 2h before RT treatment. **B**, **C** Tumor growth of L3^RR^ (**B**) and CMT^RR^ (**C**) mice treated with RT and/or pICR and/or ceralasertib. **D** Waterfall plot showing the tumor volume change of L3^RR^-bearing mice at day 18, compared to day 9 (start of treatments). **E** Diagram showing the experimental procedure of a transwell chemotaxis assay and quantification of the number of splenocytes migrated through the membrane in the different conditions tested. **F** Flow cytometry plots showing the increased number of splenocytes stained with CellTrace Violet tracker, isolated from L3^RR^+C+pICR+RT mice, compared with L3^RR^ mice. **G** Graphical diagram of the experiment to study the CXCR3-CXCL9 blockade and representation of the data showing significant inhibition of splenocytes migration in the L3^RR^+C+pICR+RT group, in presence or not of inhibitors (αCXCR3+αCXCL9/10). **H** Schematic representation showing the proposed mechanism underlying radioresistance and radioimmune-resensitization of RR tumors. Scheme created with BioRender.com. Statistical significance was defined as *p*<0.05 (*), *p*<0.01 (**) and *p*<0.001 (***)

We also sought to investigate in vivo the direct targeting of tumor cells with the ATRi ceralasertib, together with stimulation of the innate immune system, locally, with pICR. The therapeutic combination between ceralasertib+pICR + RT (hereafter referred to as “C+pICR + RT”) was tested to determine whether this strategy could further impact on tumor growth (Fig. 7A-D). As previously shown, tumor volumes in RR-tumor bearing mice treated with pICR + RT were significantly reduced, confirming the effectiveness of this therapeutic approach (Fig. 7B). Notably, in the L3^RR^ model, the C+pICR + RT combination led to further significant reduction in tumor volume (~ 60% reduction compared to controls). Volume of these tumors was also significantly smaller than that of the pICR + RT group (Fig. 7D and Fig. S9E, F). In contrast, in the CMT^RR^ model, this treatment resulted in a non-statistically significant decrease in tumor volume compared with pICR + RT (Fig. 7C).

Body weight and behavior were monitored throughout the experiment to evaluate potential signs of toxicity, and no adverse effects were detected (Fig. S10A, B). Histological analysis of spleens and kidneys, as well as measurement of liver transaminases in blood revealed lack of toxicity (Fig. S10C-E).

To explore whether pICR + RT and C+pICR + RT treatments could modulate the migration of splenocytes to the tumor, a transwell migration in vitro assay was performed. Splenocytes isolated from C+pICR + RT-treated L3^RR^ tumor-bearing mice exhibited a significantly enhanced migratory capacity towards tumor-derived stimuli compared with splenocytes from control, RT-treated or pICR-treated mice (Fig. 7E, F). To assess the contribution of the CXCR3/CXCL9 axis to this chemoattractant effect, neutralizing antibodies against CXCR3 and CXCL9 were used. Importantly, blockade of this axis significantly impaired splenocyte migration in 5 out of 6 mice, as evidenced by a marked reduction in their ability to cross the membrane toward the treated tumor cells (Fig. 7G).

A schematic representation of the proposed radioimmune-resensitization effect in the RR models induced by our therapeutic approach is shown in Fig. 7H. RR tumors developed stable resistance to DNA damage with increase in ATR signaling in cancer cells, as well as reduced immunogenicity (higher M2-like macrophages and T regulatory cells, and lower IFN-I and CXCL9/10 signaling). ATR inhibition and stimulation of IFN-I-mediated signaling increase CD8^+^ CD4^+^ recruitment through CXCL9, resensitizing tumors to RT-induced cell death.

## Discussion

Clinically, approximately 50% of NSCLC patients receive RT, either with curative intent or for palliative management. Despite substantial technological and dosimetric advances, a significant proportion of irradiated patients ultimately develop resistance to RT, markedly limiting therapeutic efficacy [35]. Acquired radioresistance is generally considered a stochastic process that may emerge following repeated irradiation of local tumor sites and is thought to be driven by the progressive accumulation of genetic and epigenetic alterations [4, 36]. Over the past decades, several groups have established NSCLC RR models through repeated exposure of parental cell lines to fractionated irradiation protocols [37, 38], providing valuable experimental platforms to investigate adaptive responses to RT. However, the molecular mechanisms underlying the acquisition and maintenance of radioresistance in tumors remain incompletely understood. RT is known to modulate the TME, promoting anti-tumor immune responses that contribute to cancer cell elimination. However, they may also include adaptive changes that reshape the TME toward an immunosuppressive phenotype, ultimately facilitating tumor immune evasion and treatment resistance [39]. To address this clinically relevant challenge, we have developed two murine isogenic RR models that provide a robust platform to investigate the contribution of the immune system to the acquisition of radioresistance. The resistant phenotype was first validated both in vitro and in vivo, confirming the stability and reproducibility of these models.

Our findings demonstrate extensive transcriptional reprogramming in the L3^RR^ model, highlighting profound alterations in the DDR network associated with the acquisition of radioresistance. RNA-seq analyses of parental L3 and L3^RR^ cells, either untreated or exposed to irradiation in vitro, revealed a basal downregulation of a substantial subset of DDR-related genes in RR cells relative to their parental counterparts. This transcriptional remodeling was accompanied by a ~ 24% increase in TMB in RR cells, suggesting that chronic adaptation to radiation may promote genomic mutations with tolerability to DNA damage. Consistent with this observation, transcriptomic analyses showed that pathways related to DDR, DNA replication, and cell cycle checkpoints were downregulated at baseline in L3^RR^ cells. Strikingly, despite this basal attenuation, irradiation robustly induced γH2AX activation in both RR models, indicating efficient recognition of DNA DSBs and rapid engagement of DDR machinery upon radiation exposure. Under these conditions, we also found significant upregulation of DNA replication and cell cycle checkpoint transcriptomic changes following RT. Furthermore, both RR models exhibited radiation-induced phosphorylation of ATR and CHK1, supporting activation of the ATR/CHK1 signaling axis as a central adaptive response to genotoxic stress. Indeed, pharmacologic inhibition of ATR using VE-821 or ceralasertib in vitro resulted in a marked radiosensitizing effect in both RR models. Induction of DNA DSBs by RT represents the primary mechanism underlying radiation-mediated cell death. Consequently, therapeutic strategies targeting DDR pathways have emerged as one of the most promising approaches to overcome RT resistance. Supporting this concept, Xu and colleagues [40] demonstrated that ATR inhibition enhances RT-induced type I IFN responses. Multiple clinical trials are currently evaluating the efficacy of DDR inhibitors in combination with RT (i.e. NCT04550104) [41].

In the present study, we demonstrated that both RR models exhibited complete resistance to RT when inoculated into immunocompetent mice. However, when tumors were implanted in immunosuppressed mice (athymic nude), both RR models showed sensitivity to RT, highlighting the critical role of the immune system in the acquisition of radioresistance in vivo. Supporting these observations, irradiated RR tumors were enriched in immunosuppressive cell populations, including Treg cells, M2-like macrophages, and PMN-MDSC. Using 3D co-culture TImS, we further demonstrated the essential contribution of Treg cells and M2-like macrophages to the development of radioresistance when co-cultured with L3 and CMT parental cell lines. Depletion of immunosuppressive myeloid populations using anti-CSF1R or anti-GR1 antibodies significantly restored radiosensitivity in vivo, directly implicating these populations as mediators of immune-driven radioresistance.

In contrast, our study shows that effector lymphocyte populations were markedly diminished, as evidenced by significant reductions in tumor-infiltrating CD8^+^ T cells, PD-1^+^ cells, and CD8^+^PD-1^+^ subsets in RR tumors, with an even more pronounced depletion after RT. CD4^+^ T cells also exhibited a declining trend, indicating a global impairment of adaptive immune surveillance. This depletion of cytotoxic lymphocytes is particularly relevant, as effective RT responses critically depend on intact innate and adaptive immune activation, including type I interferon signaling and cGAS/STING-mediated sensing of radiation-induced DNA damage [42]. Functional depletion experiments demonstrated that the cooperative activity of CD4^+^ and CD8^+^ T cells was essential for RT efficacy, highlighting the central role of adaptive immunity in mediating therapeutic responses. These functional findings were further supported by transcriptomic analyses demonstrating suppression of innate and adaptive immune signaling pathways, including IFNγ signaling, cGAS/STING activation, and cytokine networks required for cytotoxic lymphocyte recruitment, such as CXCL9 and CXCL10, which likely represents a key mechanism facilitating immune escape and resistance to RT. Collectively, these findings demonstrate that radioresistance in vivo is not solely driven by tumor cell-intrinsic mechanisms but is critically dependent on the establishment of an immunosuppressive microenvironment that impairs immune surveillance, limits RT-induced immune activation, and promotes tumor persistence despite radiation exposure.

The role of macrophages in the establishment of an immunosuppressive TME and in protecting radioresistant cancer cells from irradiation-mediated cell death is particularly intriguing. This protective role has been previously reported, although the underlying mechanisms have not been fully elucidated. The release of secreted prosurvival factors by irradiated macrophages, which can act on cancer cells (e.g., through the TNF-α/VEGF and CCL2/CCR2 axes), has been shown to contribute to this protective effect [29–31]. However, in our RR models, neither whole macrophage-conditioned supernatant nor CCL2 alone showed a protective effect on irradiated cells. It is then possible that signaling mediated by direct macrophage-cancer cell contact may play a role in this process. Indeed, direct contact between macrophages and myeloma cells has been demonstrated to be required for full protection against chemotherapy-induced apoptosis [43]. Direct macrophage-cancer cell interactions are also highly relevant to tumor progression. In prostate cancer, tumor cells interact with M2 macrophages through NOTCH1 signaling, promoting proliferation and metastasis [44]. Similarly, direct interactions between glioma cells and macrophages activate M-CSFR–STAT3 signaling, leading to tumor progression [45]. A similar mechanism could operate in the interaction between radioresistant lung cancer cells and macrophages and further investigation is warranted to elucidate the molecular mechanisms underlying this putative shielding effect.

The present work also shows that ATR expression associates with an immunosuppressive phenotype, in NSCLC. Consequently, ATR inhibition in our experimental models reduces immunosuppression and facilitates immune infiltration, likely mediated by CXCL9 and CCL2, both of them increased by ceralasertib treatment. Ceralasertib has a deleterious effect on CD8^+^ T lymphocytes, as shown by Hardaker et al., [33] and verified in our study. However, those authors demonstrated a rebound effect during the off-drug period, with increased activation and cytotoxicity ability. Moreover, ceralasertib potentiated the effect of immune checkpoint inhibitors, resulting in a strong anticancer response [33]. All this suggests that ATR could represent a relevant target to revert radioresistance in NSCLC, acting at least in part through the immune system.

Our group previously demonstrated that treatment with poly(I:C) reprograms the TME toward a proinflammatory phenotype through activation of innate immune pathways [13]. In the present study, we show that poly(I:C), either alone or in combination with ceralasertib, functions as a radiosensitizing strategy, resulting in reduction in the volume of RR tumors and a significant increase in tumoral expression of cytotoxic activation markers. Plasma cytokine analyses revealed elevated levels of the immune-recruiting chemokines CXCL9 and CXCL10 in the combination treatment groups compared with control or radiotherapy-alone-treated mice. Functional transwell assays further demonstrated that the CXCR3/CXCL9-10 axis is required for the recruitment of splenocytes in treated tumors.

CXCL9 and CXCL10 are ELR-negative CXC chemokines that bind to the CXCR3 receptor and generally exert anti-tumor effects by recruiting cytotoxic lymphocytes (CTLs), as well as NK, NKT cells, and macrophages [34]. Melvyn and colleagues [46] demonstrated a critical role for the CXCR3/CXCL9 axis in promoting the proliferation and activation of intratumoral CD8⁺ T cells following PD-1 blockade, mediated by CXCL9 derived from CD103^+^ dendritic cells in a murine colon adenocarcinoma model. In NSCLC, intratumoral administration of CXCL10 increased infiltration of CD8⁺ T cells, CD4⁺ T cells, and NK cells, resulting in tumor suppression [47]. Similarly, Raymond et al., [48] reported that intratumoral injection of dendritic cells overexpressing CXCL9 and CXCL10 improved outcomes in several NSCLC models. This effect was mediated by enhanced CD4⁺ and CD8⁺ T-cell responses and was dependent on the CXCR3 axis.

## Conclusions

In conclusion, our data demonstrate that combining innate immune stimulation and ATR inhibition with radiotherapy effectively overcomes radioresistance, an effect that is mediated by increased levels of CXCL9, responsible for the recruitment of effector proinflammatory cells. Based on our findings, a rational therapeutic strategy to improve radiotherapy efficacy in NSCLC patients with acquired radioresistance would involve a multimodal approach that simultaneously targets tumor-intrinsic DDR and reverses tumor-mediated immune suppression. Activation of innate immune pathways using TLR3-7/8 agonists could critically stimulate secretion of CXCL9-10 and recruitment or proinflammatory immune populations, improving the therapeutic efficacy. 

## Supplementary Information

Supplementary Material 1.

## Data Availability

All raw data generated in this study are available upon request from the corresponding author. Data generated or analyzed during this study are included in the main text or the supplementary materials of this article. The bulk RNA-seq data generated in this study will be publicly available in Gene Expression Omnibus (GEO). Radioresistant cells are available upon request to the senior authors.

## Abbreviations

ATR: Ataxia telangiectasia and Rad3-related
cGAS: cyclic GMP-AMP synthase
CXCL9: C-X-C motif chemokine ligand 9
DSB: DNA double-strand breaks
DAMP: Damage-associated molecular pattern
ICD: Immunogenic cell death
MDSC: Myeloid-derived suppressor cells
NSCLC: Non-small cell lung cancer
RT: Radiotherapy
RR: Radioresistant
STING: Stimulator of INterferon Genes
TLR: Toll-like receptors
TME: Tumor microenvironment

## References

[1] Dohopolski M, Gottumukkala S, Gomez D, Iyengar P. Radiation Therapy in Non-Small-Cell Lung Cancer. Cold Spring Harb Perspect Med. Cold Spring Harb Perspect Med. 2021;11(10):a037713. 10.1101/cshperspect.a037713 PMC8485739 34127511

[2] Li T, Ma W, Al-Obeidi E. Evolving Precision First-Line Systemic Treatment for Patients with Unresectable Non-Small Cell Lung Cancer. Cancers (Basel). Cancers (Basel). 2024;16(13):2350. 10.3390/cancers16132350 PMC11240640 39001412

[3] Taugner J, Käsmann L, Eze C, Rühle A, Tufman A, Reinmuth N, et al. Real-world prospective analysis of treatment patterns in durvalumab maintenance after chemoradiotherapy in unresectable, locally advanced NSCLC patients. Invest New Drugs Invest New Drugs. 2021;39:1189–96. 10.1007/s10637-021-01091-9 PMC8280025 33704621

[4] Vyfhuis MAL, Rice S, Remick J, Mossahebi S, Badiyan S, Mohindra P, et al. Reirradiation for locoregionally recurrent non-small cell lung cancer. J Thorac Dis J Thorac Dis. 2018;10:S2522–36. 10.21037/jtd.2017.12.50 PMC6123190 30206496

[5] Kalman NS, Weiss E, Walker PR, Rosenman JG, . Local Radiotherapy Intensification for Locally Advanced Non–small-cell Lung Cancer – A Call to Arms. Clin Lung Cancer. 2018;19:17–26. Elsevier Inc. 10.1016/j.cllc.2017.05.024 28712978

[6] Zhao C, Luo S, Shao Q, Li P, Huang L, Meng L, Multidisciplinary Digital Publishing Institute (MDPI). Mechanisms Underlying Radioresistance and Reversal Strategies in Non-Small Cell Lung Cancer. Int J Mol Sci. 2025;26:6559. 10.3390/ijms26146559 PMC12294630 40724808

[7] Guo S, Yao Y, Tang Y, Xin Z, Wu D, Ni C et al. Radiation-induced tumor immune microenvironments and potential targets for combination therapy. Signal Transduct Target Ther 2023 81. Nature Publishing Group; 2023;8:1–22. 10.1038/s41392-023-01462-z PMC10199044 37208386

[8] Zhou T, Zhang LY, He JZ, Miao ZM, Li YY, Zhang YM, et al. Review: Mechanisms and perspective treatment of radioresistance in non-small cell lung cancer. Front Immunol Front Media S A. 2023;14:1133899. 10.3389/fimmu.2023.1133899 PMC9971010 36865554

[9] Wang L, Li S, Zhu X. Construction of Radiation Surviving/Resistant Lung Cancer Cell Lines with Equidifferent Gradient Dose Irradiation. Dose-Response. 2020;18:1559325820982421. SAGE Publications Inc. 10.1177/1559325820982421 PMC7758662 33424518

[10] Shi Z, Hu C, Zheng X, Sun C, Li Q. Feedback loop between hypoxia and energy metabolic reprogramming aggravates the radioresistance of cancer cells. Exp Hematol Oncol Exp Hematol Oncol. 2024;13(1):55. 10.1186/s40164-024-00519-1 PMC11110349 38778409

[11] Freire J, Ajona D, de Biurrun G, Agorreta J, Segura V, Guruceaga E, et al. Silica-induced chronic inflammation promotes lung carcinogenesis in the context of an immunosuppressive microenvironment. Neoplasia (United States). 2013;15:913–24. 10.1593/neo.13310 PMC3730043 23908592

[12] Bleau AM, Freire J, Pajares MJ, Zudaire I, Anton I, Nistal-Villan E, et al. New syngeneic inflammatory-related lung cancer metastatic model harboring double KRAS/WWOX alterations. Int J cancer Int J Cancer. 2014;135:2516–27. 10.1002/ijc.28574 24473991

[13] Anfray C, Mainini F, Digifico E, Maeda A, Sironi M, Erreni M, et al. Intratumoral combination therapy with poly(I:C) and resiquimod synergistically triggers tumor-associated macrophages for effective systemic antitumoral immunity. J Immunother Cancer. 2021;9:1–17. 10.1136/jitc-2021-002408 PMC8449972 34531246

[14] Franks LM, Layton MG. Ultrastructural tumour differentiation and organ specificity in high and low metastatic lines from a mouse lung carcinoma. Br J Cancer Nat Publishing Group. 1984;49:423–9. 10.1038/bjc.1984.68 PMC1976762 6324837

[15] León S, Quintas ST, Otegui N, Redrado M, Aristu JJ, Oliveira MJ, et al. Protocol to develop a 3D mouse autologous lung tumor-immune spheroid co-culture model to advance radiotherapy-based treatments. STAR Protoc. 2025;6(3):104021. 10.1016/j.xpro.2025.104021 PMC12361748 40811056

[16] Redin E, Otegui N, Santos M, Leon S, Redrado M, Serrano D, et al. Dasatinib Remodels the Tumor Microenvironment and Sensitizes Small Cell Lung Cancer to Immunotherapy. Cancer Res Cancer Res. 2025;85:3910–29. 10.1158/0008-5472.CAN-24-2772 40712061

[17] Bolger AM, Lohse M, Usadel B. Trimmomatic: A flexible trimmer for Illumina sequence data. Bioinformatics. 2014;30:2114–20. 10.1093/bioinformatics/btu170 PMC4103590 24695404

[18] Dobin A, Davis CA, Schlesinger F, Drenkow J, Zaleski C, Jha S, et al. STAR: Ultrafast universal RNA-seq aligner. Bioinf Bioinf. 2013;29:15–21. 10.1093/bioinformatics/bts635 PMC3530905 23104886

[19] Liao Y, Smyth GK, Shi W. featureCounts: an efficient general purpose program for assigning sequence reads to genomic features. Bioinf Bioinf. 2014;30:923–30. 10.1093/bioinformatics/btt656 24227677

[20] Frankish A, Carbonell-Sala S, Diekhans M, Jungreis I, Loveland JE, Mudge JM, et al. GENCODE: reference annotation for the human and mouse genomes in 2023. Nucleic Acids Res. Volume 51. Oxford University Press; 2023. pp. D942–9. 10.1093/nar/gkac1071 PMC9825462 36420896

[21] R Core Team. (2021) R A Language and Environment for Statistical Computing. R Foundation for Statistical Computing, Vienna. - References - Scientific Research Publishing. Available from: https://www.scirp.org/reference/referencespapers?referenceid=3131254. Cited 2025 Apr 29.

[22] Robinson MD, McCarthy DJ, Smyth GK. edgeR: A Bioconductor package for differential expression analysis of digital gene expression data. Volume 26. Bioinformatics: Oxford University Press; 2009. pp. 139–40. 10.1093/bioinformatics/btp616 PMC2796818 19910308

[23] Ritchie ME, Phipson B, Wu D, Hu Y, Law CW, Shi W, et al. Limma powers differential expression analyses for RNA-sequencing and microarray studies. Nucleic Acids Res. Volume 43. Oxford University Press. 2015;43(7):e47. 10.1093/nar/gkv007 PMC4402510 25605792

[24] Yu G, Wang L-G, Han Y, He Q-Y. clusterProfiler: an R Package for Comparing Biological Themes Among Gene Clusters. OMICS. 2012;16(5):284-7. 10.1089/omi.2011.0118 PMC3339379 22455463

[25] Subramanian A, Tamayo P, Mootha VK, Mukherjee S, Ebert BL, Gillette MA, et al. Gene set enrichment analysis: a knowledge-based approach for interpreting genome-wide expression profiles. Proc Natl Acad Sci U S A Proc Natl Acad Sci U S A. 2005;102:15545–50. 10.1073/pnas.0506580102 PMC1239896 16199517

[26] Moon Y, Kim YH, Kim JK, Hong CH, Kang EK, Choi HW, et al. Evaluation of false positive and false negative errors in targeted next generation sequencing. Genome Biol. 2025;26(1):409. 10.1186/s13059-025-03882-2 PMC12670792 41327433

[27] Robinson JT, Thorvaldsdóttir H, Winckler W, Guttman M, Lander ES, Getz G, et al. Integr Genome Viewer Nat Biotechnol. 2011;29:24–6. 10.1038/nbt.1754 PMC3346182 21221095

[28] Pu Y, Ji Q. Tumor-Associated Macrophages Regulate PD-1/PD-L1 Immunosuppression. Front Immunol Front Media S A. 2022;13:874589. 10.3389/fimmu.2022.874589 PMC9110638 35592338

[29] Pinto AT, Machado AB, Osório H, Pinto ML, Vitorino R, Justino G, et al. Macrophage Resistance to Ionizing Radiation Exposure Is Accompanied by Decreased Cathepsin D and Increased Transferrin Receptor 1 Expression. Cancers (Basel). 2022;15(1):270. 10.3390/cancers15010270 PMC9818572 36612268

[30] Meng Y, Beckett MA, Liang H, Mauceri HJ, Van Rooijen N, Cohen KS, et al. Blockade of tumor necrosis factor alpha signaling in tumor-associated macrophages as a radiosensitizing strategy. Cancer Res Cancer Res. 2010;70:1534–43. 10.1158/0008-5472.CAN-09-2995 PMC8849568 20145121

[31] Pathikonda S, Tian L, Arava CM, Cheng SH, Lam YW. Radiation-induced rescue effect on human breast carcinoma cells is regulated by macrophages. Biochem Biophys Rep Elsevier B V. 2025;41:101936. 10.1016/j.bbrep.2025.101936 PMC11850746 40007574

[32] Kalbasi A, Komar C, Tooker GM, Liu M, Lee JW, Gladney WL, et al. Tumor-Derived CCL2 Mediates Resistance to Radiotherapy in Pancreatic Ductal Adenocarcinoma. Clin Cancer Res Clin Cancer Res. 2017;23:137–48. 10.1158/1078-0432.CCR-16-0870 PMC5195913 27354473

[33] Hardaker EL, Sanseviero E, Karmokar A, Taylor D, Milo M, Michaloglou C, et al. The ATR inhibitor ceralasertib potentiates cancer checkpoint immunotherapy by regulating the tumor microenvironment. Nat Commun 2024 151. Nat Publishing Group. 2024;15:1–20. 10.1038/s41467-024-45996-4 PMC10894296 38402224

[34] Tokunaga R, Zhang W, Naseem M, Puccini A, Berger MD, Soni S, W.B. Saunders. CXCL9, CXCL10, CXCL11/CXCR3 axis for immune activation – A target for novel cancer therapy. Cancer Treat Rev. 2018;63:40–7. 10.1016/j.ctrv.2017.11.007 PMC5801162 29207310

[35] Alhaddad L, Pustovalova M, Blokhina T, Chuprov-Netochin R, Osipov AN, Leonov S. Ir-surviving nsclc cells exhibit different patterns of molecular and cellular reactions relating to the multifraction irradiation regimen and p53-family proteins expression. Cancers (Basel). MDPI AG. 2021;13(11):2669. 10.3390/cancers13112669 PMC8198560 34071477

[36] Sato K, Shimokawa T, Imai T. Difference in Acquired Radioresistance Induction Between Repeated Photon and Particle Irradiation. Front Oncol Front Media S A. 2019;9:1213. 10.3389/fonc.2019.01213 PMC6863406 31799186

[37] Li H, Zhang J, Yu B, Yang T, Liu B, Li F, et al. RSPO3 regulates the radioresistance of Non-Small cell lung cancer cells via NLRP3 Inflammasome-Mediated pyroptosis. Radiother Oncol. Elsevier Ireland Ltd. 2024;200:110528. 10.1016/j.radonc.2024.110528 39245068

[38] Wang S, Li Z, Li P, Li L, Liu Y, Feng Y, et al. SOX2 Promotes Radioresistance in Non-small Cell Lung Cancer by Regulating Tumor Cells Dedifferentiation. Int J Med Sci Ivyspring Int Publisher. 2023;20:781. 10.7150/ijms.75315 PMC10198148 37213675

[39] Venkatesulu BP, Mallick S, Lin SH, Krishnan S. A systematic review of the influence of radiation-induced lymphopenia on survival outcomes in solid tumors. Crit Rev Oncol Hematol Elsevier. 2018;123:42–51. 10.1016/j.critrevonc.2018.01.003 29482778

[40] Feng X, Tubbs A, Zhang C, Tang M, Sridharan S, Wang C et al. ATR inhibition potentiates ionizing radiation-induced interferon response via cytosolic nucleic acid-sensing pathways. EMBO J. 2020;39(14):e104036. 10.15252/embj.2019104036 PMC7361286 32484965

[41] Dillon MT, Guevara J, Mohammed K, Patin EC, Smith SA, Dean E, et al. Durable responses to ATR inhibition with ceralasertib in tumors with genomic defects and high inflammation. J Clin Invest Am Soc Clin Invest. 2024;134:e175369. 10.1172/JCI175369 PMC10786692 37934611

[42] Chen J, Wang S, Ding Y, Xu D, Zheng S. Radiotherapy-induced alterations in tumor microenvironment: metabolism and immunity. Front Cell Dev Biol Front Media SA. 2025;13:1568634. 10.3389/fcell.2025.1568634 PMC12066526 40356601

[43] Zheng Y, Cai Z, Wang S, Zhang X, Qian J, Hong S, et al. Macrophages are an abundant component of myeloma microenvironment and protect myeloma cells from chemotherapy drug-induced apoptosis. Blood Blood. 2009;114:3625–8. 10.1182/blood-2009-05-220285 PMC2766678 19710503

[44] Shi F, Sun MH, Zhou Z, Wu L, Zhu Z, Xia SJ, et al. Tumor-associated macrophages in direct contact with prostate cancer cells promote malignant proliferation and metastasis through NOTCH1 pathway. Int J Biol Sci Int J Biol Sci. 2022;18:5994–6007. 10.7150/ijbs.73141 PMC9682538 36439868

[45] Komohara Y, Horlad H, Ohnishi K, Fujiwara Y, Bai B, Nakagawa T, et al. Importance of direct macrophage-tumor cell interaction on progression of human glioma. Cancer Sci Cancer Sci. 2012;103:2165–72. 10.1111/cas.12015 PMC7659278 22957741

[46] Chow MT, Ozga AJ, Servis RL, Frederick DT, Lo JA, Fisher DE, et al. Intratumoral Activity of the CXCR3 Chemokine System Is Required for the Efficacy of Anti-PD-1 Therapy. Immun Cell Press. 2019;50:1498–e15125. 10.1016/j.immuni.2019.04.010 PMC6527362 31097342

[47] Shinn CK, Saddawi-Konefka R, Salanga CL, Schokrpur S, Gutkind JS, Handel TM. Activating the CXCR3/CXCL10 pathway overrides tumor immune suppression by enhancing immune trafficking and effector cell priming in head and neck squamous cell carcinoma. bioRxiv Prepr Serv Biol. 2025.04.24.650529.

[48] Lim RJ, Salehi-Rad R, Tran LM, Oh MS, Dumitras C, Crosson WP et al. CXCL9/10-engineered dendritic cells promote T cell activation and enhance immune checkpoint blockade for lung cancer. Cell Reports Med. Cell Press. 2024;5(4):101479. 10.1016/j.xcrm.2024.101479 PMC11031384 38518770

