# Supplementary material for: CXCL9-driven immune rewiring and tumor-intrinsic ATR signaling inhibition restore radiotherapy response in lung cancer
Source: J Exp Clin Cancer Res. 2026 Sep 22;45:218. doi: 10.1186/s13046-026-03834-z (PMC13670451; doi:10.1186/s13046-026-03834-z)
Supplement: Supplementary file 1 [file 13046_2026_3834_MOESM1_ESM.docx]

**Table S1.** Sequences of primers used for gene expression quantification in qPCR.

| Gene | Sequence forward (5' 🡪 3') | Sequence reverse (3' 🡪 5') |
| --- | --- | --- |
| Hprt | TCCTCCTCAGACCGCTTTT | CCTGGTTCATCATCGCTAAT |
| Ccl2 | CAGGTCCCTGTCATGCTTCT | GAGTGGGGCGTTAACTGCAT |
| Cxcl9 | CGAGGCACGATCCACTACAA | AGGCAGGTTTGATCTCCGTT |
| Cxcl10 | GATGACGGGCCAGTGAGAAT | TCAACACGTGGGCAGGATAG |
| Sting1 | GAACACCGGTCTAGGAAGCA | TAGTCCAAGTTCGTGCGAGG |
| Prf1 | TTGGTGGGACTTCAGCTTTCC | CCATACACCTGGCACGAACT |
| GrB | GAAGCCAGGAGATGTGTGCT | GCACGTTTGGTCTTTGGGTC |
| Ifng | TCAAGTGGCATAGATGTGGAAGAA | TGGCTCTGCAGGATTTTCATG |

**Table S2.** Specifications of primary antibodies used for western blotting.

| Antibody (Reference) | Specie | Dilution |
| --- | --- | --- |
| β-actin (Sigma, A5441) | Mouse | 1:10000 |
| γH2AX (CST, 9718) | Rabbit | 1:1000 |
| Akt (CST, 9272) | Rabbit | 1:1000 |
| ATR (CST, 13934) | Rabbit | 1:500 |
| cGAS (CST, 79978) | Rabbit | 1:1000 |
| Chk1 (CST, 2360S) | Mouse | 1:1000 |
| ERK1/2 (CST, 91012) | Rabbit | 1:1000 |
| p38 (CST, 9212) | Rabbit | 1:1000 |
| p53 (Santa Cruz, sc-126) | Mouse | 1:1000 |
| P70S6K (CST, 2708) | Rabbit | 1:1000 |
| p-Akt (Ser473) (CST, 9271) | Rabbit | 1:1000 |
| p-ATR (Ser428) (CST, 2853S) | Rabbit | 1:500 |
| p-Chk1 (Ser345) (CST, 2348D) | Rabbit | 1:1000 |
| p-ERK1/2 (Thr202/Tyr204) (CST, 4370) | Rabbit | 1:1000 |
| p-p38 (Thr180/Tyr182) (CST, D3F9) | Rabbit | 1:1000 |
| p-p53 (Ser 15) (CST, 9284) | Rabbit | 1:1000 |
| p-P70S6K (Thr421/Ser424) (CST, 9204S) | Rabbit | 1:1000 |
| p-STING (CST, 72971) | Rabbit | 1:1000 |
| p-TBK1 (CST, 5483) | Rabbit | 1:1000 |
| STING (CST, 13647) | Rabbit | 1:1000 |
| TBK1 (CST, 3504) | Rabbit | 1:1000 |

**Table S3.** Fluorochrome-conjugated antibodies and dilutions used for immunophenotyping by flow cytometry.

| Antibody / Clone | Fluorochrome | Dilution | Reference | Company |
| --- | --- | --- | --- | --- |
| CD11b / M1/70 | BV510 | 1:400 | 101245 | BioLegend |
| CD11c / N418 | APC | 1:80 | 117310 | BioLegend |
| CD19 / 1D3 | BV661 | 1:200 | 612971 | BioLegend |
| CD206 / C068C2 | BV650 | 1:30 | 141723 | BioLegend |
| CD25 / PC61 | PerCP | 1:100 | 102027 | BioLegend |
| CD4 / RM4-5 | BV395 | 1:400 | 740208 | BioLegend |
| CD45 / 30.F11 | APC-H7 | 1:500 | 103154 | BioLegend |
| CD8 / 53-6.7 | BV510 | 1:400 | 100751 | BioLegend |
| F4/80 / BM8 | BV421 | 1:20 | 123132 | BioLegend |
| FC Block / 2.4G2 | - | 1:100 | 553142 | BioLegend |
| FoxP3 / 3G3 | PECy7 | 1:100 | ab210232 | Abcam |
| GrB / QA16A02 | PE | 1:100 | 372207 | BioLegend |
| Ly6C / HK1.4 | AlexaF488 | 1:400 | 128006 | BioLegend |
| Ly6G / 1A8 | PerCP | 1:400 | 127654 | BioLegend |
| MHC-II / M5/114.15.2 | PECy7 | 1:300 | 107630 | BioLegend |
| NK1.1 / PL136 | APC | 1:20 | 108710 | BioLegend |
| Nkp46 / 29A1.4 | BV650 | 1:20 | 137635 | BioLegend |
| PD-1 / 29F.1A12 | BV421 | 1:80 | 135218 | BioLegend |
| PD-L1 / 10F.9G2 | PE | 1:40 | 124308 | BioLegend |
| ViaKrome 808 | IR885 | 1:100 | C36628 | Beckman |

**Table S4.** List of antibodies and conditions used for IHC

| Antibody (Reference) | Dilution | Antigen retrieval | Specie |
| --- | --- | --- | --- |
| Gr1 (BioLegend, 108402) | 1:1500 | - | Rat |
| PD-1 (CST, 86163) | 1:100 | Tris-EDTA, pH 9 | Rabbit |
| CD8 (CST, 98941) | 1:500 | Citrate, pH 6 | Rabbit |
| F4/80 (CST, 70076) | 1:1000 | Tris-EDTA, pH9 | Rabbit |

**Table S5.** Antibodies, opals and dilutions used for multiplex immunofluorescence

| Antibody (Reference) | Dilution | Antigen retrieval | Opal / Dilution |
| --- | --- | --- | --- |
| mPD-L1 (CST, 64988) | 1:400 | Tris-EDTA, pH 9 | 780 / 1:25 |
| PD-1 (CST, 84651) | 1:100 | Tris-EDTA, pH 9 | 570 / 1:600 |
| FoxP3 (CST, 12653) | 1:600 | Citrate, pH 6 | 480 / 1:100 |
| CD4 (ab183685) | 1:400 | Citrate, pH 6 | 520 / 1:300 |
| CD8a (CST, 98941) | 1:500 | Citrate, pH 6 | 620 / 1:100 |
| F4/80 (CST, 70076) | 1:400 | Citrate, pH 6 | 570 / 1:600 |
| Arg1 (CST, 93668) | 1:200 | Tris-EDTA, pH 9 | 620 / 1:100 |
| CD86 (CST, 19589) | 1:300 | Citrate, pH 6 | 690 / 1:100 |

**Table S6.** Gene Ontology (GO) categories, normalized enrichment score (NES) and adjusted p-value corresponding to transcriptomic profiles comparing L3^RR^ cells with L3 cells.

| Category of Biological process | NES | Adj. p.val |
| --- | --- | --- |
| REACTOME CELL CYCLE | -2.54 | 4.8E-56 |
| REACTOME CELL CYCLE MITOTIC | -2.61 | 1.2E-52 |
| HALLMARK G2M CHECKPOINT | -2.83 | 3.8E-38 |
| REACTOME CELL CYCLE CHECKPOINTS | -2.63 | 1.2E-32 |
| REACTOME DNA REPLICATION | -2.49 | 1.4E-17 |
| REACTOME MITOTIC SPINDLE CHECKPOINT | -2.53 | 5.4E-17 |
| REACTOME CHROMOSOME MAINTENANCE | -2.42 | 1.1E-13 |
| KEGG CELL CYCLE | -2.36 | 4.0E-13 |
| REACTOME DNA REPLICATION PRE INITIATION | -2.34 | 8.1E-12 |
| KEGG DNA REPLICATION | -2.45 | 6.9E-10 |
| REACTOME ACTIVATION OF ATR IN RESPONSE TO REPLICATION STRESS | -2.43 | 1.5E-09 |
| REACTOME DNA DOUBLE STRAND BREAK REPAIR | -2.15 | 2.9E-09 |
| HALLMARK MITOTIC SPINDLE | -2.00 | 4.8E-09 |
| REACTOME DNA REPAIR | -1.87 | 2.1E-08 |
| WP DNA REPAIR PATHWAYS FULL NETWORK | -2.03 | 3.8E-07 |
| KEGG MISMATCH REPAIR | -2.23 | 7.1E-06 |
| REACTOME BASE EXCISION REPAIR | -2.12 | 7.6E-06 |
| HALLMARK DNA REPAIR | -1.87 | 1.0E-05 |
| KEGG NUCLEOTIDE EXCISION REPAIR | -1.76 | 1.3E-02 |

**Table S7.** Gene Ontology (GO) categories, normalized enrichment score (NES) and adjusted p-value corresponding to transcriptomic profiles comparing non-irradiated L3^RR^ tumors with non-irradiated L3 tumors.

| Category of Biological process | NES | Adj. p.val |
| --- | --- | --- |
| REACTOME INTERFERON GAMMA SIGNALING | -2.28 | 1.2E-06 |
| BROWNE INTERFERON RESPONSIVE GENES | -2.14 | 1.1E-04 |
| WP PATHWAYS OF NUCLEIC ACID METABOLISM AND INNATE IMMUNE SENSING | -1.66 | 6.8E-02 |
| WP DNA REPAIR PATHWAYS FULL NETWORK | 1.50 | 1.8E-02 |
| REACTOME DNA DOUBLE STRAND BREAK RESPONSE | 1.78 | 1.1E-02 |
| REACTOME BASE EXCISION REPAIR | 1.83 | 6.3E-03 |
| REACTOME DNA DOUBLE STRAND BREAK REPAIR | 1.87 | 1.7E-04 |
| REACTOME MISMATCH REPAIR | 1.98 | 6.7E-03 |
| KEGG DNA REPLICATION | 2.20 | 1.8E-04 |
| REACTOME ACTIVATION OF ATR IN RESPONSE TO REPLICATION STRESS | 2.37 | 1.2E-05 |

**Table S8.** Gene Ontology (GO) categories, normalized enrichment score (NES) and adjusted p-value corresponding to transcriptomic profiles comparing L3^RR^+RT tumors with L3+RT tumors.

| Category of Biological Process | NES | Adj. p.val |
| --- | --- | --- |
| REACTOME DNA REPLICATION | 2.51 | 7.9E-12 |
| REACTOME STABILIZATION OF P53 | 2.19 | 6.3E-05 |
| REACTOME ACTIVATION OF ATR IN RESPONSE TO REPLICATION STRESS | 1.92 | 3.7E-03 |
| REACTOME INTERLEUKIN 1 SIGNALING | 1.77 | 3.1E-03 |
| REACTOME INTERLEUKIN 12 SIGNALING | 1.74 | 2.9E-02 |
| REACTOME DNA REPAIR | 1.55 | 3.1E-03 |
| REACTOME INTERLEUKIN 15 SIGNALING | -1.75 | 4.0E-02 |
| KEGG JAK STAT SIGNALING PATHWAY | -1.80 | 1.4E-03 |
| KEGG NATURAL KILLER CELL MEDIATED CYTOTOXICITY | -1.81 | 3.4E-03 |
| REACTOME INTERLEUKIN 2 FAMILY SIGNALING | -1.82 | 1.8E-02 |
| ISHIKAWA STING SIGNALING | -1.83 | 1.4E-02 |
| WP REGULATORY CIRCUITS OF THE STAT3 SIGNALING PATHWAY | -1.88 | 3.8E-03 |

**Fig. S1.** **A.** Representative diagram showing the generation of L3^RR^ and CMT^RR^ models, from their isogenic control cells. **B.** Representation of the scheme used to deplete CD4^+^, CD8^+^, CSFR1^+^ or GR1^+^ cells *in vivo*. Images created with [BioRender.com](http://biorender.com).

**Fig. S2.** Example of the gating strategy to study the lymphoid cell populations by flow cytometry.

**Fig. S3.** Example of the gating strategy to study the myeloid cell populations by flow cytometry.

**Fig. S4.** **A** Validation of radioresistance *in vivo* was performed in mice bearing CMT and CMT^RR^ tumors. Mice received irradiation at 8 Gy per day for five consecutive days (5×8 Gy). The response observed was consistent with that obtained using the selected radiotherapy regimen (5×5 Gy). **B.** PD-L1 levels in L3 or L3^RR^ cells, stimulated or not with IFNγ **C.** Induction of M2-polarization by the conditioned media obtained from L3^RR^+RT cells compared to that of L3+RT cells. **D.** Induction of T regulatory cells by the conditioned media obtained from L3^RR^+RT cells compared to that of L3+RT cells. **E.** Western blots to show p-ERK1/2, ERK1/2, p-AKT and AKT protein levels. β-actin was used as loading control. L3, L3^RR^, CMT and CMT^RR^ were kept without irradiation (Ctrl) or irradiated with 6 Gy. **F.** Apoptosis induced by ceralasertib in L3 or L3^RR^ cells, treated or not with RT. **G-J.** Percentage of cell proliferation of L3, L3^RR^, CMT or CMT^RR^ cells, with or without irradiation, in the presence or absence of different doses of the P38 inhibitor Ralimetinib dimesylate or the P70S6K inhibitor PF-4708671. Statistical significance was defined as p<0.05 (*), p<0.01 (**) and p<0.001 (***).

**Fig. S5.** **A.** Gene Ontology (GO) categories significantly altered in L3^RR^ cells, compared to L3 cells. Many categories related to DDR, as well as cell proliferation are found in the analysis. **B.** Tumor mutational burden (TMB) of L3 and L3^RR^ cells. Analysis was conducted by whole exome sequencing (WES), BWA (BWA/0.7.17-foss-2018b), VarScan2 (VarScan/2.4.2-Java-1.8) and Mutect2 (GATK/4.0.8.1- foss-2018b-Python-2.7.15) algorithms. **C-D.** MTS proliferation assay of L3 and L3^RR^ cells (C), and CMT and CMT^RR^ cells (D), 24h, 48h or 72h after plating. **E.** GSEA analysis of the categories “DNA replication” and “Cell cycle checkpoint”. Negative enrichment is appreciated in L3^RR^ when compared to L3. On the contrary, positive enrichment is found when L3^RR^ cells are treated with RT.

**Fig. S6. A.** Protein levels of pATR/ATR, cGAS, pSTING/STING, pTBK1/TBK1 and γH2AX, in L3 or L3^RR^ untreated or treated with the ATRi ceralasertib, RT or the combination ceralasertib+RT. **B.** Schematic representation summarizing the protocol to prepare 3D co-cultures resulting in tumor spheroids (TSp) and tumor-immune spheroids (TImS). **C.** Representative images of L3 TImS on day 1. Tumor cells are seen in blue (CellTrace^TM^ Violet Cell Proliferation), M2-like macrophages in red (CellTrace^TM^ Far Red Cell Proliferation) and T regulatory cells in green (CellTrace^TM^ CFSE Cell Proliferation). Scale bar: 100 μm. **D.** mRNA levels of CCL2 in irradiated macrophages. Statistical significance was defined as p<0.05 (*), p<0.01 (**) and p<0.001 (***).

**Fig. S7. A.** Representative images of GR1^+^ cells in L3, L3^RR^, L3+RT and L3^RR^+RT tumors (scale bar: 50 μm). **B.** Flow cytometry analysis showing a trend toward an increased percentage of PMN-MDSC (CD45^+^CD11b^+^Ly6G^high^Ly6C^low^) in the CMT^RR^ and CMT^RR^+RT tumors. **C.** Flow cytometry for Treg cell infiltration in L3, L3^RR^, CMT and CMT^RR^ treated or untreated tumors. **D.** Representative images of IHQ and quantification of CD8^+^ cells in L3, L3^RR^, L3+RT, L3^RR^+RT tumors. Scale bar: 50 μm. **E.** Representative multiplex immunofluorescence (mIF) images of PD-1^+^, FOXP3^+^, CD8^+^, F4/80^+^, PD-L1^+^, CD4^+^ markers. Scale bar: 20 μm. **F.** Representative images of IHQ and quantification of PD-1^+^ cells in L3, L3^RR^, L3+RT, L3^RR^+RT tumors. Scale bar: 50 μm. **G.** Flow cytometry analysis of CD8^+^PD-1^+^ tumor infiltration in L3, L3^RR^, L3+RT and L3^RR^+RT tumors. **H.** Flow cytometry analysis of CD8^+^PD-1^+^ infiltration in CMT, CMT+RT, CMT^RR^ and CMT^RR^+RT tumors. **I.** PCA and Volcano plot analyses showing the diversity in the transcriptomic profiles of L3, L3+RT, L3^RR^ and L3^RR^+RT tumors and the significant changes in gene expression between L3^RR^ and L3 tumors. The red color represents genes whose log2FC <-1 and p-value <0.05 or log2FC >1 and p-value <0.05. The blue color represents genes whose log2FC >-1 and p-value <0.05 or log2FC <1 and p-value <0.05. The green color represents genes whose log2FC <-1 and p-value >0.05 or log2FC >1 and p-value >0.05. Statistical significance was defined as p<0.05 (*), p<0.01 (**) and p<0.001 (***).

**Fig. S8. A.** Tumor volume in mice bearing L3^RR^ cells, untreated or treated with the ATRi ceralasertib, RT or the combination of both. **B.** Tumor infiltration of CD8^+^ T lymphocytes and F4/80+ macrophages in ceralasertib-treated L3^RR^-tumor bearing mice. **C.** CXCL9 and CCL2 levels in L3 and L3^RR^ cells, exposed or not to ceralasertib. **D.** Treg cell differentiation from CD4+ lymphocytes, cultured with conditioned media of L3 or L3^RR^ cells exposed or not to RT or RT+ATRi. **E.** Macrophage polarization cultured with conditioned media of L3 or L3^RR^ cells exposed or not to RT or RT+ATRi. **F.** mRNA expression of cytokines in irradiated CMT^RR^ tumors, compared to untreated tumors. **G.** mRNA levels of CXCL9 in the L3^RR^ cells with CXCL9 overexpression (L3^RR^-CXCL9 OE). **H.** Number of migrated splenocytes (in a transwell assay) towards L3^RR^-Scr cells (controls) or L3^RR^-CXCL9 OE cells. **I-K.** Infiltration of CD45^+^ (I), CD11b^+^ (J) and PMN-MDSC (K) cells in L3^RR^-CXCL9 OE or L3^RR^-Scr (control) tumors. Statistical significance was defined as p<0.05 (*), p<0.01 (**) and p<0.001 (***).

**Fig. S9. A.** Graphical representation of mice weight at the start (S) and end (E) of treatments in the L3^RR^, L3^RR^+RT, L3^RR^+pIC and L3^RR^+RT+pIC groups. **B.** The same representation for CMT^RR^, CMT^RR^+RT, CMT^RR^+pIC and CMT^RR^+RT+pIC groups. **C.** Analyses of total macrophages (F4/80^+^) shows an increase in both RR tumors upon combination of RT+pICR (p<0.05 for CMT^RR^+pICR+RT compared to CMT^RR^ or CMT^RR^+RT). **D.** Plasma levels of IL1β, CXCL16, IL25, CCL2 and IL6 in L3+RT, CMT+RT and L3^RR^ or CMT^RR^ treated with RT alone or in combination of pICR. **E.** Tumor volumes of the L3^RR^ model at day 18, in untreated (controls) or treated with RT, pICR+RT or C+pICR+RT. **F.** Spaghetti plots showing the individual growth of each tumor from the experiment shown in Fig. 7B. Statistical significance was defined as p<0.05 (*), p<0.01 (**) and p<0.001 (***).

**Fig. S10. A.** Graphical representation of weight in L3^RR^-tumor bearing mice at the start (S) and end (E) of treatments: RT, pIC+RT and C+pIC+RT. “C” stands for ceralasertib. **B.** The same representation for CMT^RR^-tumor bearing mice. **C.** Representative histological images of kidney and spleen to assess toxicity of the pICR+RT and C+pICR+RT groups of treatment. Scale bar: 25 μm. **D-E.** Hematological analysis of liver transaminases shows no differences in the levels of ALT and AST in the C+pICR+RT group when compared with control mice. D: L3^RR^ mice; E: CMT^RR^ mice. Statistical significance was defined as p<0.05 (*), p<0.01 (**) and p<0.001 (***).
